# Ultra‐high‐performance supercritical fluid chromatography‐mass spectrometry for the analysis of organic contaminants in sediments

**DOI:** 10.1002/jssc.202200668

**Published:** 2022-11-10

**Authors:** Josephine S. Lübeck, Jan H. Christensen, Giorgio Tomasi

**Affiliations:** ^1^ Department of Plant and Environmental Sciences Analytical Chemistry Group University of Copenhagen Frederiksberg Denmark

**Keywords:** D‐optimal design, nontarget screening, sediment analysis, supercritical fluid chromatography

## Abstract

A nontarget screening method was developed based on D‐optimal designs for ultra‐high performance supercritical fluid chromatography with positive and negative electrospray ionization mode mass spectrometry. A mixture of organic contaminants such as pesticides, steroids, surfactants, phenolic and fatty acids, and polycyclic aromatic hydrocarbon derivatives, was used for the optimization. An aprotic mixture of dichloromethane and acetone [3:1] performed overall best as the injection solvent. The highest peak capacities (*n*) were accomplished at the shallowest gradient (1%B/min), ammonium formate (*n* = 378 in negative ionization mode), or ammonium acetate (*n* = 327 in positive ionization mode) in methanol as the modifier. Capillary voltage, make‐up solvent flow rate, water, and additive concentration were the most significant factors for improving peak intensity: higher peak intensities were obtained at lower additive concentrations (5mM ammonium formate), and with 5% water in positive ionization mode. Conversely, water had detrimental effects in negative ionization mode. The optimized method was used to quantify organic contaminants in 17 freshwater sediment samples from Copenhagen, Denmark. Out of 50 monitored contaminants, 35 were detected in at least one sample. Further, the method has a potential for target and nontarget screening analysis of organic contaminants in solid matrices.

**Article Related Abbreviations**
Add: modifier additive type
AmAc: ammonium acetate
AmFo: ammonium formate
CapV: capillary voltage
ConeV: cone voltage
DCM: dichloromethane
FA: formic acid
g: gradient slope
MTBE: tert‐butyl methyl ether
muF: make‐up solvent flow rate
QC: quality control
THF: tetrahydrofuran
w: water addition
[Add]: modifier additive concentration
[w]: modifier water concentration

## 1 INTRODUCTION

Freshwater sediments are greatly affected by anthropogenic activities as a result of industrial and household waste, wastewater effluents, or road runoff. They become a sink for a wide variety of low‐ to mid‐polarity organic contaminants and their degradation products [1, 2]. Nonpolar compounds such as polycyclic aromatic hydrocarbons, polychlorinated biphenyls, and other persistent organic pollutants are routinely monitored in solid matrices using target workflows with GC‐MS or GC coupled to electron capture detectors [2, 3, 4, 5, 6]. Midpolar to polar compounds such as certain pharmaceuticals, and household or personal care products can be analyzed with UHPLC and high‐resolution MS [2, 6, 7] to avoid the use of derivatization in GC and allow the determination of non‐volatile and thermally labile compounds.

Ultra‐high performance supercritical fluid chromatography (UHPSFC) is complementary to LC and GC utilizing normal‐ and reverse‐phase columns and sub‐ or supercritical CO_2_ as the mobile phase in combination with a modifier, for example, methanol [8]. Pharmaceuticals, chiral separation, and bioanalysis have been the main applications of UHPSFC [9, 10]; it has also been applied to analyze complex matrices, for example, unconventional fuels [9, 11–15], natural products [16, 17] and environmental samples [18, 19].

Nontarget screening aims to give a more comprehensive picture of a sample's chemical composition than targeted workflows, where no reference standards or limited a priori information on the type of compounds in a sample are available [20]. Several nontarget screening studies have been performed on wastewater [21, 22] and aquatic environmental samples [23, 24, 25]. To analyze a broad range of organic contaminants in a nontarget screening, a high peak capacity and sensitive detection are essential; both are affected by a wide range of factors (e.g., stationary phase chemistry, mobile phase composition, and various ionization parameters) that need optimization.

This study aimed to develop a UHPSFC‐ESI‐MS method for target and nontarget screening analysis of sediment samples. Two D‐optimal designs were performed, one focussing on the peak capacity of the UHPSFC separation, and the second focussing on the peak intensity in the MS detection. D‐optimal designs can straightforwardly handle incomplete domains, discrete levels for continuous factors, and multiple‐level categorical variables in one model [13, 26–30]. The method was tested on 17 freshwater sediment samples from Copenhagen, Denmark. To the best of our knowledge, this is the first UHPSFC‐ESI‐MS method for target and nontarget screening of sediment samples.

## 2 MATERIALS AND METHODS

### 2.1 Chemicals, standards, and samples

#### 2.1.1 Chemicals

Methanol and ethanol, both LC‐MS grade, Chromasolv; tetrahydrofuran (THF, ≤ 250ppm butylated hydroxytoluene), anhydrous tert‐butyl methyl ether (MTBE) and ammonium formate (AmFo) were purchased from Sigma‐Aldrich (St. Louis, MO, USA). Acetone, hexane, and dichloromethane (DCM), HPLC grade, were acquired from Rathburn Chemicals (Walkerburn, Scotland). MilliQ water was obtained in‐house with a type I ultrapure water purification system from ELGA‐Veolia LabWater (High Wycombe, UK). Formic acid and ammonia (25% solution), both LiChropur, and ammonium acetate (AmAc) were purchased from Merck KGaA (Darmstadt, Germany).

#### 2.1.2 Standards

A mixture of 60 analytical standards (nominal concentration of approximately 100ppb per analyte) was prepared by mixing three primary standard solutions: 33μl of each solution with a concentration between 28 and 40ppm, filled up to 10ml with DCM:acetone [3:1] (Tables S1 and S2).

#### 2.1.3 Samples

Sediment samples (0–30cm) were collected from a lake (Utterslev Mose) and an adjacent fortress channel in Copenhagen, Denmark in 2017. Details about the sampling site and sample preparation – a sequential pressurized liquid extraction – are described [31]. Briefly, the extraction entailed two steps, (i) with methanol:water [1:1] and (ii) with DCM:acetone [3:1] to extract a large range of compounds with varying polarities.

### 2.2 Instrumentation and constant method conditions

An Acquity UPC^2^ hyphenated to a G2‐Si Synapt HRMS from Waters (Milford, MA, USA) was used. The separation was performed on a Torus DIOL column (3.00×100mm, 1.7μm) with a VanGuard pre‐column (diol, 2.1×5.0mm, 1.7μm) from Waters. The choice of the column was based on literature [12, 13]. The post‐column splitter interface from Waters was used to connect the UPC^2^ and MS, with a make‐up solvent flow via a quaternary solvent manager (Waters). Methanol as a modifier (B‐solvent), column temperature (40°C), automated backpressure (140bar), flow rate (1.5ml/min), and injection volume (3μl) were kept constant. Strong and weak washing solvents were methanol:water [4:1] and isopropanol, respectively. Both positive (ESI^+^) and negative (ESI^−^) ionization modes were applied. If not stated otherwise, the MS parameters were set as follows: source temperature 120°C, desolvation temperature 600°C, desolvation gas flow 1000L/h, cone gas flow 100L/h, scan rate 0.2scans/s, scan type ‘centroid’, and mass range 50–1200Da. Lockspray calibration with leucine‐enkephalin (0.2ng/ml, Waters) was performed every 30s. The data was acquired with MassLynx; TargetLynx (v4.1; Waters) and R software (RStudio 2022.02) were used to process the data.

### 2.3 Method development

#### 2.3.1 Injection solvent screening

To investigate whether a solvent exchange would increase the solubility or improve the ionization efficiency of the target analytes, five solvent combinations were tested: DCM:acetone [3:1], THF, hexane, MTBE, and a mixture of DCM:acetone:hexane [3:1:4]. The peak height of the parent ion in each ionization mode was averaged (triplicate analysis) and normalized to the largest measured response between the tested injection solvents. The method conditions are summarised in Table S3.

#### 2.3.2 D‐optimal design I: chromatography

Three factors were investigated to maximize the peak capacity: two qualitative (water addition and modifier additive type) and one quantitative factor (gradient steepness) (Table 1). These factors have an effect on the peak width, the number of eluted peaks in the given run time, and the interactions between the compounds and mobile and stationary phases, respectively [8, 12–14]. The R‐based CAT software (Group of Chemometrics, Italian Chemical Society) was used to set up and assess the experimental design. The model consists of eight coefficients (*cf*. Section S2, Table S4). The investigated additives were 0.1% formic acid (FA), 20mM AmFo, and 20mM AmAc in methanol. Water was either added volumetrically (5%) to the modifier or not. The gradient steepness was set to 1.0, 2.5, or 4.0%B/min, the total run time was 40 min (Figure S1). The make‐up solvent (flow rate: 0.1ml/min) was the same as the tested modifier additive. Ionization parameters were: capillary voltage 1.5kV, cone voltage (ConeV) 30V, and desolvation temperature 500°C.

**TABLE 1:** Factors of both D‐optimal designs

		Level		
	Acronym	Low	Centre	High
*Design I (Chromatography)*				
Modifier additive type^a^, ^b^	**Add**	0.1% FA	20mM AmFo	20mM AmAc
Water addition (%)^a^	**w**	0	‐	5
Gradient steepness (%B/min)	**g**	1.0	2.5	4.0
*Design II (Ionisation)*				
Capillary voltage (kV)	**CapV**	1.5	2.25	3.0
Cone Voltage (V)	**ConeV**	20	25	30
Make‐up flow rate (ml/min)	**muF**	0.1	0.15	0.2
Make‐up solvent type (10mM)^a^	**muPH**	FA	‐	NH_3_
Water concentration in modifier (%)	**[w]**	0.0	2.5	5.0
Modifier additive concentration (mM)	**[Add]**	5.0	12.5	20.0

^a^ Qualitative factors.

^b^ Modifier additive was set as either no additive (pure methanol as reference) or the specified additive (FA, AmFo, or AmAc).

The response in *Design I* was peak capacity, calculated as the sum over the different isocratic and gradient regions of the analysis time, and corrected for the number of missing analytes that were not detected (Equation (1) [12]):

(1) $$\begin{array}{ccc} n_{\mathrm{corr}} & = & \left[\frac{{\bar{N}}^{0.5}}{4}\ln\left(\frac{t_{L}^{\left(I\right)}}{t_{0}^{\left(I\right)}}\right)+\frac{t_{G}}{{\bar{w}}_{0.5}}+\frac{{\bar{N}}^{0.5}}{4}\ln\left(\frac{t_{L}^{\left(\mathrm{II}\right)}}{t_{0}^{\left(\mathrm{II}\right)}}\right)\right]c_{\mathrm{miss}}^{-1}\\ & & \equiv n_{\mathrm{sum}}c_{\mathrm{miss}}^{-1} \end{array}$$

with *n*_sum_ the uncorrected peak capacity, $\bar{N}$ as the average column plate number [12], $t_{L}^{x}$ the retention time of the last‐eluting analyte in the first isocratic hold (I), gradient or second isocratic hold (II); *t_0_* the dead time of the particular region; *t_G_* the gradient time; ${\bar{w}}_{0.5}$ is the average full peak width at half maximum. The correction factor $c_{miss}^{-1}$ was defined as $c_{\mathrm{miss}}^{-1}=\left(1+a_{\mathrm{miss}}/a_{\mathrm{total}}\right)$, for *a*_miss_ as the maximum number of missing and *a*_total_ as the total number of analytes.

#### 2.3.3 D‐optimal design II: ionization

*Design II* focussed on the ionization efficiency based on capillary voltage (CapV), cone voltage (ConeV), make‐up solvent flow rate (muF), make‐up solvent type (muPH), the concentration of additive ([Add]), and concentration of water ([w]). The levels of the six factors were based on literature [13, 26, 32] (Table 1). The model is comprised of 27 coefficients (*cf*. Section S2, Table S5).

*Design II* was based on results from *Design I*, namely that AmFo and 1.0%B/min provided overall the best peak capacity in both ionization modes. The run time in *Design II* was decreased to 28min; the new gradient program was: 3.0min hold at 1.0%B, gradient to 20%B in 19min, to 30%B in 1.0min, hold for 1.5min, and return to 1.0%B in 0.5min and equilibrate for 3.0min. Other chromatographic conditions were unchanged.

As a response in *Design II*, the average of the range‐scaled (0 to 1, where 1 is the best) peak areas across all analytes and across all experiments ( ${\bar{A}}_{\mathrm{sc}}$) was used (Equation (2)):

(2) $${\bar{A}}_{\mathrm{sc}}=\frac{1}{N}\sum_{j}\frac{A_{\mathrm{ij}}-m_{j}}{M_{j}-m_{j}}$$

where $A_{ij}$ is the peak area for the *ith* experiment and the *jth* compound; *N* is the number of analytes; m_j_ and M_j_ the minimum and maximum peak area across all experiments for the *j^th^* analyte, respectively. Only analytes that were detected in all experiments were included.

The model was validated with an additional set of experiments (Table S5). In all of these, 5mM AmFo was used as an additive, the make‐up flow rate was 0.1ml/min and the make‐up solvent additive was 10mM FA. CapV and ConeV were set to 1.5kV and 20V, and 3kV and 30V for ESI^−^ and ESI^+^, respectively. All water concentrations (0%, 2.5%, and 5%) were tested. Three experiments were added from the original experimental domain, including the center point experiment 248.

#### 2.3.4 Quality control

Facilitator samples with a shorter gradient and pure methanol as a modifier (Table S6) were run frequently along the sequence per ionization mode as quality control (QC); a total of 28 QCs. These facilitator samples contained the same selected organic contaminants and were used to correct for changes in the ionization efficiency and sensitivity of the MS detector (*cf*. below). Several blanks (DCM:acetone [3:1]) were run between different additive types and concentrations to flush and equilibrate the column. The sequence in *Design II* was blocked according to additive concentration. Nine randomly chosen replicates (accounting for 25% of the data set) were added to obtain better estimates of the experimental variation.

#### 2.3.5 Data treatment

Peak areas were corrected before range‐scaling with a piece‐wise linear correction function between the facilitator samples, using the factors from a probabilistic quotient normalization that were calculated on the facilitator samples alone [33]. Briefly, the correction factor for the *nth* facilitator sample is calculated as the average across the number of analytes (*N*) of the ratio between the area of analyte *j*, $A_{nj}$, and the average area for the same analyte in all facilitator samples ${\bar{A}}_{j}$ (Equation (3)):

(3) $$q_{n}=N^{-1}\sum_{j=1}^{N}\frac{A_{\mathrm{nj}}}{{\bar{A}}_{j}}$$

The correction factor for the *i^th^* experiment was calculated as in Equation (4):

(4) $$q_{i}=p_{i}q_{\mathrm{prev}}+\left(1-p_{i}\right)q_{\mathrm{next}}$$

where *q*_prev_ and *q*_next_ are the correction factors for the closest preceding and following facilitator samples, and 0<p<1 is a factor that depends linearly on the distance between the sample and the two facilitator samples along the sequence (namely: $p=\frac{s_{QC_{\mathrm{next}}}-s_{i}}{s_{QC_{\mathrm{next}}}-s_{QC_{\mathrm{prev}}}}$, where *s* is the position along the sequence of the sample or facilitator sample). The same procedure was used for experiments that were added later which also included the same facilitator samples for both QC and drift correction.

${\bar{A}}_{\mathrm{sc}}$ values from the validation sets were scaled according to the range of the set on which the models were calculated. A batch correction factor was calculated based on the average corrected peak areas for all analytes (Equation (5)):

(5) $$f_{\mathrm{val},v}=N^{-1}\sum_{j}\frac{{\bar{A}}_{j}^{\left(\mathrm{cal}\right)}}{{\bar{A}}_{j}^{\left(\mathrm{val},v\right)}}$$

where ${\bar{A}}_{j}^{\left(\mathrm{cal}\right)}$ and ${\bar{A}}_{j}^{(\mathrm{val},v)}$ denote the average (within‐batch) corrected peak area for the *jth* analyte in the calibration set and the *vth* validation set, respectively.

### 2.4 Quantification

Sediment samples were analyzed with the optimal conditions in ESI^−^ and ESI^+^. Five analytical facilitator samples (no matrix, ca. 1000ppb per analyte) for drift correction (*cf*. Section 2.3.5), and matrix‐containing QCs per sampling site (three in total), unspiked and spiked (final concentration of 50ppb per analyte) in duplicates, were run in the sequence. Quantifier ions of the analytes were integrated into TargetLynx (Table S2), and qualifier ions were checked. One‐point matrix‐matched response factors were calculated by the difference between spiked and unspiked peak area and divided by the corresponding spiking concentration. Peak areas were then divided by the corresponding response factor to determine the concentrations in the samples. Subsequently, the extraction blank was subtracted from each sample.

## 3 RESULTS AND DISCUSSION

### 3.1 Injection solvent screening

Interactions between injection solvent, initial mobile phase composition, and stationary phase were shown to be significant in UHPSFC as they can negatively affect the peak shapes of early‐eluting compounds [27, 34]. Here, analytes were dissolved in five aprotic solvents or solvent mixtures, that is, DCM:acetone [3:1], THF, MTBE, hexane, and DCM:acetone:hexane [3:1:4]. Injection solvents were assessed according to average rank for the normalized peak height ($\bar{r}$), the median normalised peak height (M) and interquartile range (Table S7).

The performance of both DCM:acetone and THF were comparable in both ESI^−^ and ESI^+^, where the former was ranking highest and showing the smallest interquartile in ESI^−^ ($\bar{r}$= 1.8, M = 1.00), while THF showed slightly better $\bar{r}$ and the lowest interquartile range for ESI^+^ ($\bar{r}$= 2.5, M = 0.96). This can be explained by THF performing slightly better than DCM:acetone in dissolving some phenols and aromatic acids (Table S7). However, THF also showed more outliers compared to DCM:acetone with lower normalized peak heights, for example, for pirimicarb, imidacloprid, imazalil, and triclocarban. The significantly higher density of DCM (i.e., 1.33 vs 0.886g/ml of THF) could affect the chromatographic efficiency by creating zones of locally high elution strength [34]. However, this hypothesis was rejected due to the constant retention times of the analytes across all injection solvents.

The solvent mixture DCM:acetone:hexane and hexane had the poorest performance (M = 0.77 for both). Hexane did not sufficiently dissolve analytes such as imidacloprid, bisphenol A, and some monoaromatic acids; it showed larger variability than the other injection solvents. While the mixture DCM:acetone:hexane clearly improved the solubility of some analytes compared to hexane alone, the benefits were limited. Desfontaine et al. found that MTBE performed well at higher injection volumes (5 or 10μl) [27], but here it performed comparably mediocre (M = 0.82 and $\bar{r}$= 3.3). Moreover, a blank injection of MTBE exposed numerous background peaks potentially arising from MTBE, which can cause unwanted interferences in nontarget screening studies, thus, impair peak identification (Figure S2).

In conclusion, the solvent mixture DCM:acetone [3:1] was chosen over THF because it performed best overall and because no solvent exchange of the nonpolar sediment extracts would be necessary [31].

### 3.2 Design I – chromatography optimization

The higher the peak capacity, the more information can be extracted from complex environmental samples. *Design I* aimed at optimizing the gradient steepness (quantitative factor), the type of additive, and the addition of water (wa to the modifier (both qualitative factors).

The D‐optimal algorithm was run in the entire experimental space, that is, from eight to 45 experiments (Section S2). A selection of twelve experiments (Table S4) yielded a variance inflation factor of 1.59, which was deemed acceptable. The experimental plan was symmetric with four runs per gradient, six runs for w, and three runs per additive (e.g., 0.1% FA, 20mM AmAc, 20mM AmFo, and no additive).

In ESI^−^, three analytes (e.g., PFOS, PFOA, and diphenic acid) were excluded from the peak capacity calculation due to fluctuating retention in all experiments and carry‐over. 2‐carboxycinnamic acid did not give a response. In ESI^+^, three analytes (e.g., anisole, cypermethrin, and 1H‐benzo(g)indole) were excluded due to low retention and insufficient peak intensities. No adduct formation was observed for the undetected analytes. Analytes that were in the mixtures but do not ionize in ESI were disregarded (Table S1). Hence, 26 (out of 30) and 20 (out of 23) analytes were used to calculate peak capacities in ESI^−^ and ESI^+^ mode, respectively.

The model explains 96.3% and 57.7% of the variance for ESI^−^ and ESI^+^, respectively (Figure S3). The coefficient for gradient steepness (g) was significant in both ionization modes: the shallower the gradient, the higher the peak capacity, which is consistent with previous observations for both reversed‐phase HPLC and UHPSFC [12, 35].

**FIGURE 1:**
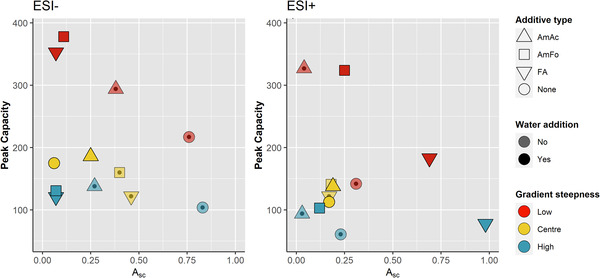
Results of *Design I*. ${\bar{A}}_{\mathrm{sc}}$ vs peak capacity. Additive: AmAc – 20mM ammonium acetate; AmFo – 20mM ammonium formate; FA – 0.1% formic acid. Gradient steepness [%B/min]: Low – 1.0; Center – 2.5; High – 4.0

Figure 1 illustrates the results of *Design I*. Although the coefficients for the type of additive were not significant (Figure S3), they are relatively large and positive in both ionization modes, indicating a positive effect of the additives on the peak capacity. Peak capacities were on average 36% (ESI^−^) and 45% (ESI^+^) higher in experiments with additives compared to experiments without additives (*cf*., Table S4). This is due to an increasing elution strength of the mobile phase with an additive present, resulting in altered retention and narrower peaks [8]. Highest peak capacities were obtained with AmFo (ESI^−^: $n_{\mathrm{corr}}=378$, ESI^+^: $n_{\mathrm{corr}}=324$), FA (ESI^−^: $n_{\mathrm{corr}}=353$) and AmAc (ESI^+^: $n_{\mathrm{corr}}=327$) (Figure 1). Furthermore, water (w) and the interaction of water and the gradient steepness (w×g) were significant in ESI^−^ but not in ESI^+^ (Figure S3).

Without correcting for missing analytes, the highest peak capacity in ESI^−^ was obtained with 0.1% FA, 5% water, and the shallowest gradient steepness ($n_{\mathrm{sum}}=441$). However, seven analytes were missing in that experiment, resulting in $n_{\mathrm{corr}}=353$ (Table S4), whereas it was only two in the same experiment with AmFo ($n_{\mathrm{corr}}=378$). In particular, several acidic analytes (e.g., adamantanecarboxylic acid and perfluorooctanoic acid) and bisphenol A were not detected in the experiments with 0.1% FA and 5% water, indicating a substantial change in the apparent pH of the mobile phase and thus, an unfavorable dissociative state of these analytes. Further, the elution strength of 0.1% FA in methanol (up to 30%B) – unlike the ammonium salts – did not suffice to elute strongly retained compounds of potential interest in environmental samples. For example, linear alkylbenzene sulphonates, which are anionic surfactants arising from anthropogenic activities [36], were detected in the sediment extracts from Copenhagen; however, they eluted first after several blank injections when using FA as an additive (data not shown).

Larger averaged peak areas for all detectable analytes (${\bar{A}}_{\mathrm{sc}}$, Equation 2) were observed without any additive (ESI^−^) or with 0.1% FA (ESI^+^), and with the steepest gradient (Figure 1). The higher response in ESI^+^ with 0.1% FA is likely due to the lower apparent pH of the mobile phase, facilitating protonation of the analytes [37]. Yet, these experiments had comparatively low peak capacities (between 78 and 217, Figure 1).

A significant decrease in ${\bar{A}}_{\mathrm{sc}}$ was observed when water was added to the modifier in ESI^−^ (${\bar{A}}_{\mathrm{sc}}$ ≤ 0.25, Figure 1). The negative effect of water in ESI^−^ has been described elsewhere [37]. Nováková et al. have shown that water in the make‐up solvent in SFC‐ESI^−^‐MS leads to a decrease in the signal intensity of doping agents by > 50% compared to no water [38]. Conversely, they described a > 150% signal enhancement in ESI^+^ with the addition of up to 20% water into the make‐up solvent compared to runs where no water was added. Higher ${\bar{A}}_{\mathrm{sc}}$ values in ESI^+^ were also observed herein, for example, with 0.1% FA and 5% water (${\bar{A}}_{\mathrm{sc}}$ ≥ 0.69, Figure 1). Since its effect on ionization efficiency is seemingly concentration‐dependent, water concentration was included as a factor in *Design II*.

${\bar{A}}_{\mathrm{sc}}$ values were consistently lower and independent of gradient steepness in experiments with 20mM of either AmAc or AmF, and with or without water present (Figure 1). That is indicative of ion suppression effects due to the relatively high concentrations of these salts. Additionally, both ammonium salts had a positive effect on peak capacities in both ionization modes, which is consistent with other findings [38].

To choose one of the ammonium salts, additional experiments were performed in ESI^+^, however, no significant difference in ${\bar{A}}_{\mathrm{sc}}$ between the two ammonium salts were found (*p*‐value > 0.05 in *t*‐test) (Table S8 and Figure S3). Thus, as AmFo performed significantly better than AmAc in terms of peak capacity in ESI^−^, it was selected as an additive and tested at varying concentrations in *Design II*.

### 3.3 Design II – ionisation optimisation

The shallowest gradient (1.0%B/min) and AmFo as additive were the basis for *Design II*. The levels for the factors in *Design II* can be found in Table 1. Out of 486 potential candidate points in the experimental domain, a design with 36 experiments with a maximum variance inflation factor of 1.43 was chosen for either ionization mode (Table S5). The response was ${\bar{A}}_{\mathrm{sc}}$ (Equation (2)) for all detectable analytes. The models described 94.4% and 88.1% explained variance in ESI^−^ and ESI^+^, respectively.

Eight analytes (e.g., 1‐adamantcarboxylic acids, 2‐carboxycinammic acid, 2‐phenylphenol, cyclohexanecarboxylic acid, pyrocatechol, diphenic acid, and salicylic acid) were excluded from the calculations because they were either too low in peak height (≤ 100 counts) or S/N (≤ 25), or because of severe retention time shifts (>0.5min) between replicate analyses (e.g., 1‐hydroxy‐2‐naphthoic acid). PFOS and PFOA have eluted in all *Design II* experiments thanks to the higher eluent strength of AmFo. Consequently, 22 out of 30 (ESI^−^), and 17 out of 23 (ESI^+^) compounds were included to calculate ${\bar{A}}_{\mathrm{sc}}$.

**FIGURE 2:**
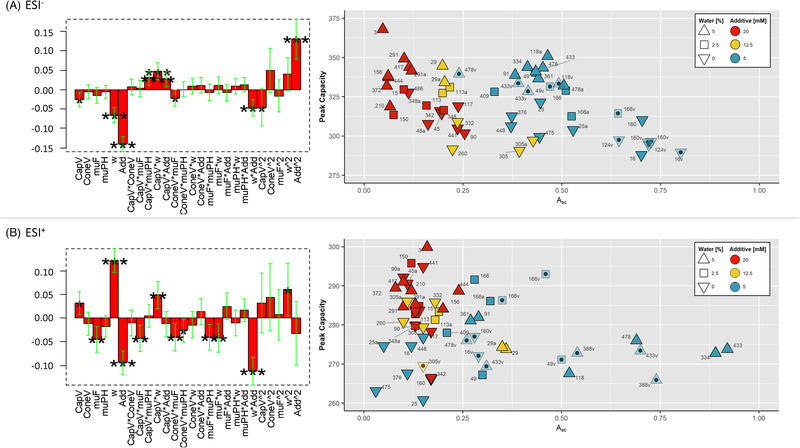
Results of *Design II* in (A) ESI^−^ and (B) ESI^+^. On the left: Coefficients of ${\bar{A}}_{\mathrm{sc}}$; CapV ‐ capillary voltage; ConeV – cone voltage; muF – make‐up solvent flow rate; muPH – make‐up solvent type; w – water concentration; Add ‐ additive concentration. On the right: ${\bar{A}}_{\mathrm{sc}}$ vs peak capacity. Transparent data points indicate validation experiments (#v), replicates specified with #a (*cf*. Table S4). The colors and shapes of the candidate points are according to the coefficients [w] and [Add].

Results for ESI^−^ and ESI^+^ are summarised in Figure 2. CapV, water concentration ([w]), and additive concentration [Add] appear to have a significant negative effect on the sensitivity in ESI^−^. The interaction between water and additive concentration ([w]×[Add]) is significant and negative in both ionization modes (Figure 2). In ESI^+^, water has a positive effect, and make‐up solvent flow (muF) is also significant (and negative) both in linear terms as well as in some interactions.

Overall, a high additive concentration (20mM) in the modifier causes a decrease in ${\bar{A}}_{\mathrm{sc}}$. Significant ion suppression effects have been linked to additive concentration in LC‐ [37] and SFC‐ESI‐MS [38]: a significant increase in sensitivity in both ESI^+^ and ESI^−^ was observed at low concentrations of AmFo (1 and 5mM) compared to medium concentration (10mM), while the response decreased at 20mM [38]. They recommend 10mM AmFo in order not to sacrifice peak shapes. Herein, 5mM AmFo was chosen as an acceptable concentration in both ionization modes, because peak shapes were overall not compromised (not shown). While the effect is clear, and the results suggest that the highest responses would be achieved without any additive, this would have a significant, detrimental effect on the peak capacity (*cf*. Section 3.2).

Water had a positive effect on the peak capacity in ESI^−^, confirming observations made in *Design I*. For example, at 20mM AmFo, peak capacities ranged from 302 (Exp 90) to 368 (Exp 3) for 0 to 5% water, respectively. The increase in peak capacity is most likely due to the higher eluent strength when water is present. Conversely, water decreased ${\bar{A}}_{\mathrm{sc}}$significantly in ESI^−^ (Figure 2), indicating a decrease in the detector sensitivity which is consistent with previous findings [37, 38]. The decrease in detector sensitivity due to water has been linked to a change in the apparent pH of the mobile phase composition (e.g., the apparent pH of a CO_2_‐methanol mixture with 2% water was found to have an acidity close to an aqueous pH of 1) [39].

The coefficient [w]×[Add] was negative and significant in both ionization modes; the range of the effect of [w] decreased with a higher AmFo concentration (Figure 2). In ESI^+^, [w] had a significant positive effect at a low additive concentration; lowering [w] at the same additive concentration decreased ${\bar{A}}_{\mathrm{sc}}$ in ESI^+^, for example, ${\bar{A}}_{\mathrm{sc}}=0.89$ (Exp 334) at 5% water vs ${\bar{A}}_{\mathrm{sc}}=0.03$ (Exp 475) at 0% water. When both 5% water and 20mM AmFo were used, ${\bar{A}}_{\mathrm{sc}}$ was largely negatively impacted.

CapV significantly affected ${\bar{A}}_{\mathrm{sc}}$, that is, a higher ${\bar{A}}_{\mathrm{sc}}$ were obtained at lower and higher capillary voltage for ESI^−^ and ESI^+^, respectively (Figure 2). These results are partially in contrast with other works indicating the importance of selecting appropriate standards for optimization: for example, a lower capillary voltage (1kV) was shown to provide higher responses for pharmaceutical compounds in ESI^+^ [26], while capillary voltage was shown not to significantly affect lignin compounds within the range of 2–3kV in ESI^−^ [13].

A negative effect of the muF on ${\bar{A}}_{\mathrm{sc}}$ was observed but appears to be significant only in ESI^+^, whereas the apparent pH of the make‐up solvent had no significant impact (except for some interactions) in either ionization mode (Figure 2). The decrease in response at higher muFs is possibly caused by a stronger dilution of the analyte concentration in the merged mobile phase (UHPSFC flow plus make‐up solvent flow) [26]. A lower muF was considered optimal also in other studies [13, 26]; however, none of them tested flow rates below 0.2ml/min. Conversely, a flow rate below 0.1ml/min could drastically deteriorate the ionization efficiency at the gradient starting conditions with high CO_2_ content, that is, without solvent, there would be no ion production in ESI.

### 3.4 Diagnostics and final method

**FIGURE 3:**
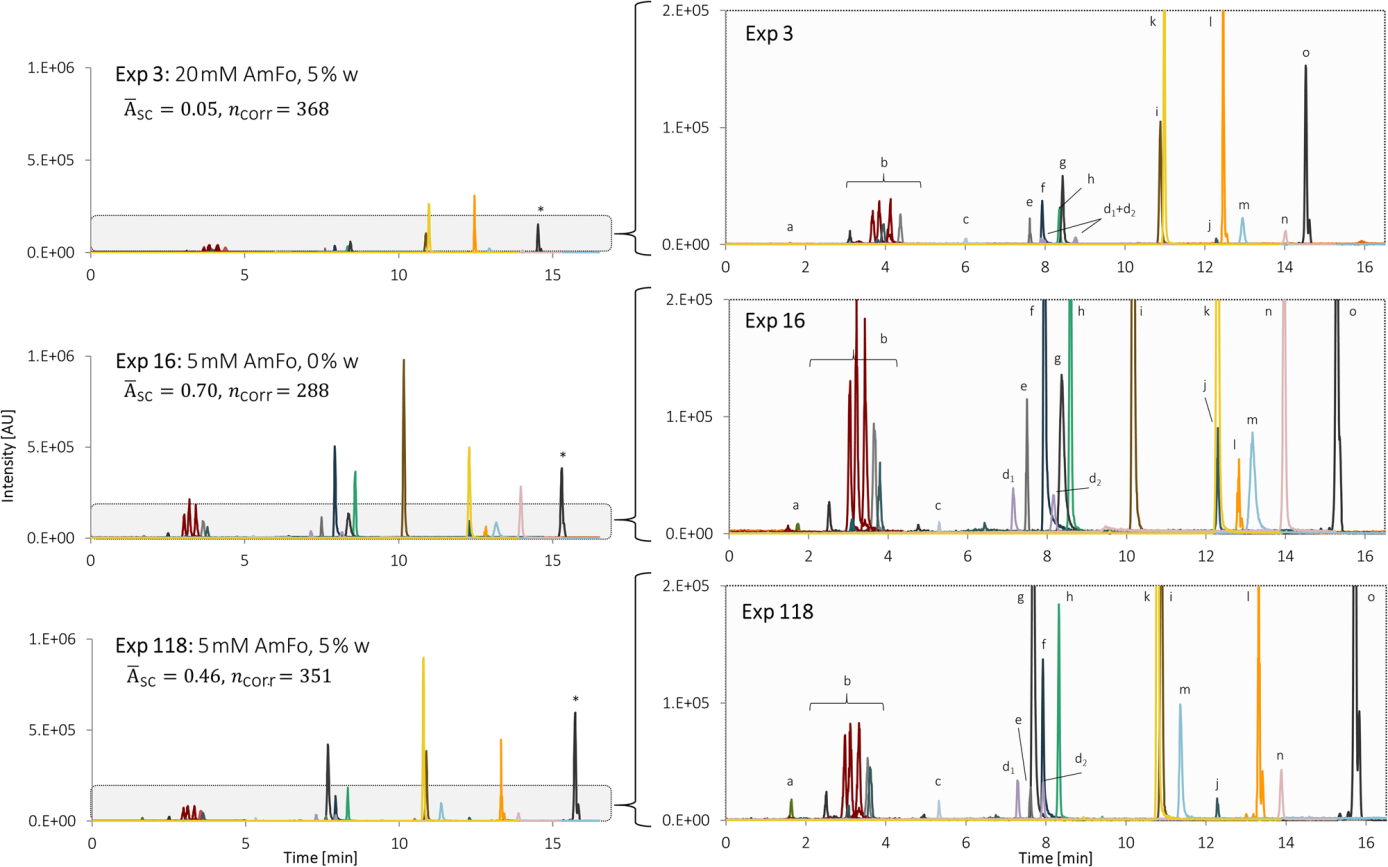
Extracted ion chromatograms of the experiments (Exp) 16, 118, and 3 of *Design II*, total (left), zoom‐in (right). Factors in common: CapV: 1.5kV, muF: 0.1ml/min. ^*)^ Scaled peak (×0.075). Peak annotations: *a* – 2‐phenylphenol; *b* – aliphatic acids, triclosan; *c* – 4‐butylbenzoic acid; *d_1/2_* – 1‐ and 2‐naphthoic acid; *e* – estrone; *f* – 2‐mercaptobenzothiazole; *g* – pentachlorophenol; *h* – 9‐hydroxyphenanthrene; *i* – imidacloprid, *j* – bisphenol A, *k –* triclocarban; *l* – PFOA ([M‐COOH]^−^ fragment: *m/z* 368.977), *m* – 1‐pyrenecarboxylic acid, *n* – 1,7‐dihydroxynaphthalene, *o* – PFOS

Figure 3 shows extracted ion chromatograms of three experiments (Exp) with varying additive and water concentrations in ESI^−^: Exp 16 (${\bar{A}}_{\mathrm{sc}}=0.70,n_{\mathrm{corr}}=288$), Exp 118 (${\bar{A}}_{\mathrm{sc}}=0.46,n_{\mathrm{corr}}=351$), and Exp 3 (${\bar{A}}_{\mathrm{sc}}=0.05,n_{\mathrm{corr}}=368$). In general, signal intensities were the lowest in Exp 3 for all analytes, demonstrating once more ion suppression at high additive (20mM AmFo) and water concentrations (5%). It also shows that the effects of [w] and [Add] were analyte‐dependent. For example, aliphatic acids (peak group *b*) in Exp 16 are approximately two to five times as high as the corresponding signals in Exp 118 and 3, respectively. Other analytes' signal intensities decreased drastically upon w, such as estrone (peak *e*), 2‐mercaptobenzothiazole (*f*), imidacloprid (*i*), bisphenol a (*j*), and 1,7‐dihydroxynaphthalene (*n*) (Figure 3). Contrarily, a sevenfold decrease in signal intensity was observed for PFOA in Exp 16 (no water) vs Exp 118 (5% water). It could be that the water creates an acidic environment as the apparent pH of the mobile phase decreases from ∼ 5–1 [39], resulting in a higher nondissociated fraction of PFOA (pKa ∼ 3.8 [40]). Additionally, water affected the elution order, retention times, and some peak widths, for example, 1‐pyrenecarboxylic acid (peak *m*) eluted 1.82min sooner, and tailing was significantly reduced from Exp 16–118; pentachlorophenol (*g*) eluted 0.7min earlier and was 2.5 times higher in the same experiments.

Predictions of ${\bar{A}}_{\mathrm{sc}}$ in ESI^−^ were satisfying with a root‐mean‐square‐error‐of‐prediction of 0.099 (Figure S5). Experiments 16, 160, and 124 showed the highest ${\bar{A}}_{\mathrm{sc}}$ (*cf*. Figure 2). These experiments only differed in the muF and ConeV, supporting the conclusion that these factors are in fact non‐significant. In positive mode, however, the root‐mean‐square‐error‐of‐prediction value was considerably higher (i.e., 0.23 – Figure S5). A generalized drop in sensitivity was observed in the validation set experiments which was partly reflected in the ${\bar{A}}_{\mathrm{sc}}$’s (e.g., in Exp 433, it dropped from 0.92 to 0.61 in the first, then to 0.31 in the second validation set – Figure 2). There is no significant difference in either average peak areas or standard deviation between facilitator samples in calibration and validation after batch correction. The experimental pooled standard deviations for ${\bar{A}}_{\mathrm{sc}}$ and the peak capacity based on nine replicates (*df* = 8) and model residuals (*df* = 18) were not significantly different in either ionization mode according to an F‐test. However, such a drop in peak areas was not observed for all compounds (i.e., it decreased for 11 and 12 out of 17 compounds in the validation sets 1 and 2, respectively – data not shown). This significantly limits the effect of batch correction which is based on an average across all compounds (Equation 5). It would be possible to correct every single compound independently, but that would not be applicable in a nontarget screening study because of the many unknowns that could not be corrected. The reason for this behavior may relate to changes in additive and water concentration over time, cleanliness of the ion source, and changes in adduct formation.

**FIGURE 4:**
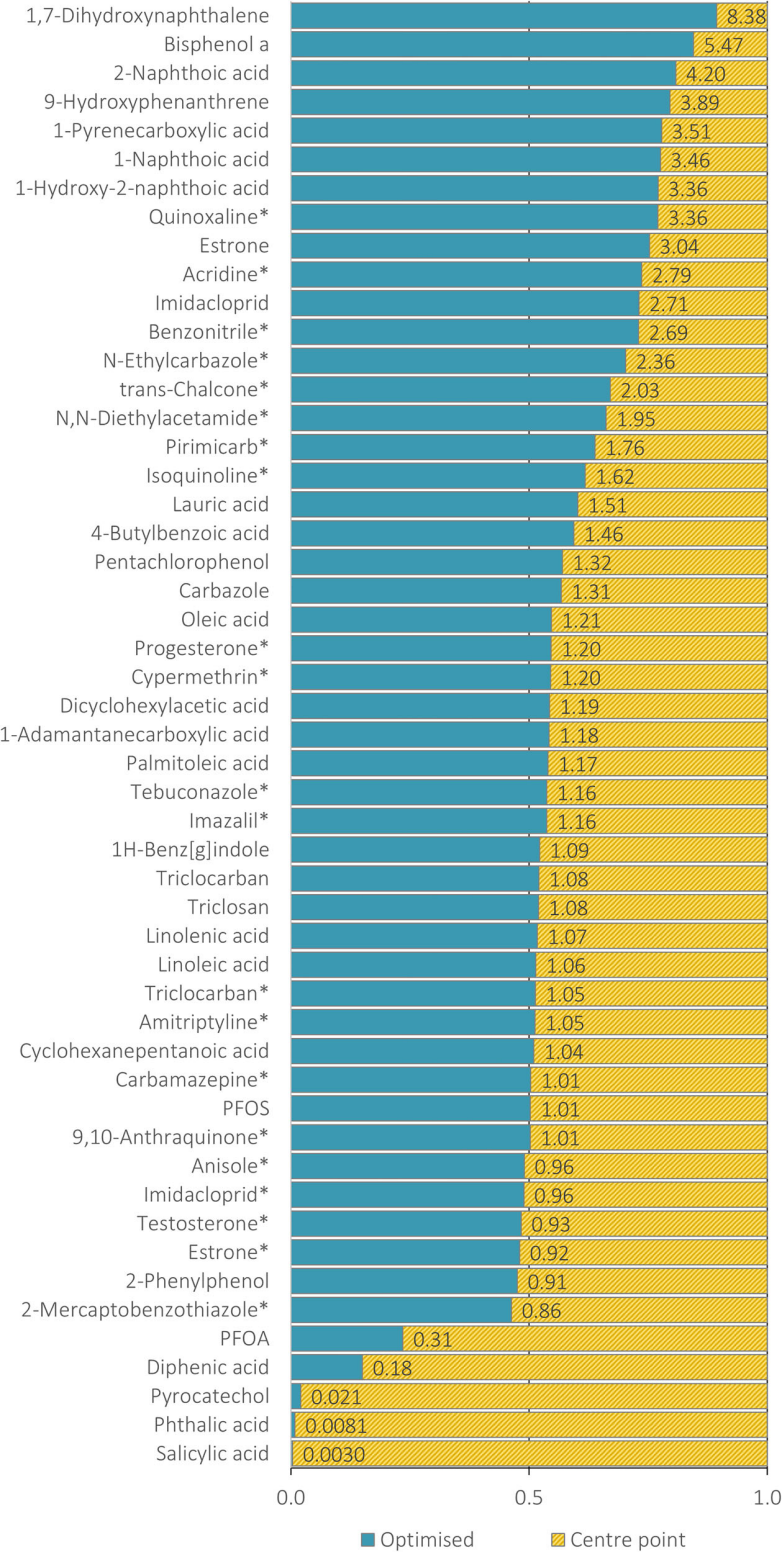
Fractional comparison of peak areas between the optimized (blue) and center point (yellow) conditions in ESI^−^ and ESI^+^ (labeled with ^*^). $\mathrm{Ratio}=\frac{A_{\mathrm{Optimised}}}{A_{\mathrm{Centre}\mathrm{point}}}$

The main focus of *Design II* was to optimize ionization efficiency and the results indicate that the effect on peak capacity, while not negligible, is considerably smaller in both positive and negative modes than for the corrected peak areas. Namely, for the experimental conditions with the highest ${\bar{A}}_{\mathrm{sc}}$ (Exp 16 and 334 for ESI^−^ and ESI^+^, respectively), the peak areas were higher for 78% of the analytes compared to the center point, Exp 248 (Figure 4); while peak capacities decreased from 343±3 to 313±10, and from 294±9 to 285±4 in ESI^−^ and ESI^+^, respectively. It is noteworthy that analytes in ESI^−^ such as PFOA, diphenic acid, salicylic acid, phthalic acid, and pyrocatechol performed considerably worse than at the center point, which is most likely related to the dissociative state at the low apparent pH due to the addition of 2.5% water at the center point (*cf*. above).

### 3.5 Analysis of sediment samples

**FIGURE 5:**
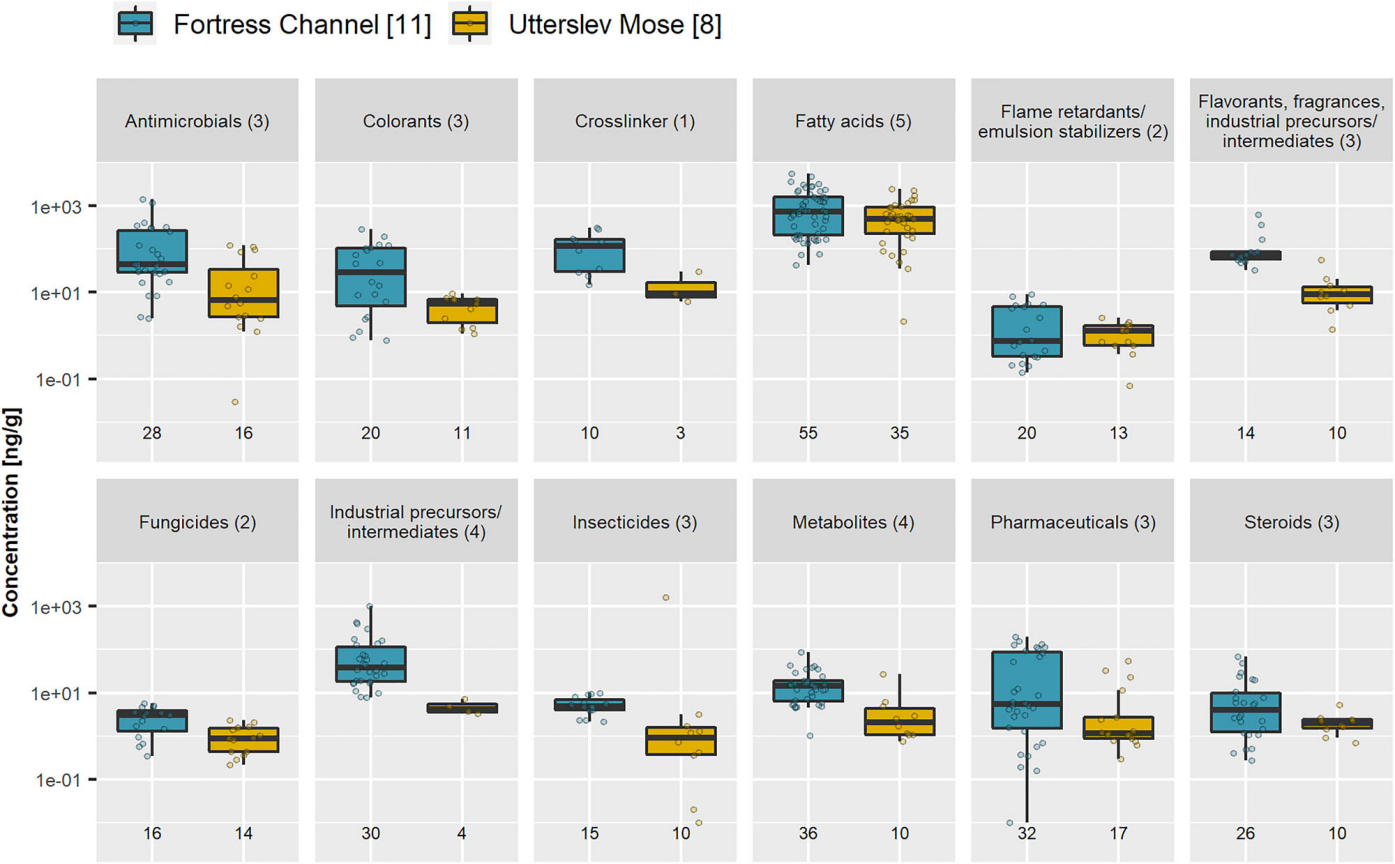
Concentrations (ng/g) of detected analytes in the samples, sorted after functional use or compound group (*cf*. Tables S2 and S9). The total number of samples per sample type is given in brackets; the total number of analytes per functional use is given in parentheses; the number of included observations is given below each box plot. Only peaks with S/N > 10 after blank subtraction were included.

Seventeen freshwater sediment samples from Copenhagen, Denmark were analyzed for the selected organic contaminants with UHPSFC‐ESI‐MS using the optimized experimental conditions (Table 2). Out of the 50 organic contaminants, 35 were observed in at least one sample (Figure 5 and Table S9). Overall, higher concentrations and relative abundance of especially industrial compounds were detected in the fortress channel compared to the adjacent lake Utterslev Mose – a nature preserve – which confirms previous findings on the same samples measured with GC×GC‐MS [31]. This is probably due to the fact that the channel has received the surrounding sewer overflow and other pollutants of anthropogenic activity for several decades. Complementary to the nonpolar compounds detected in [31], more polar analytes were found, such as fatty acids, steroids, phenolic acids, triclocarban, carbamazepine, PFOA, and PFOS. Especially bisphenol A (crosslinker), 1H‐benzo(g)indole, and compounds with an antimicrobial function such as pentachlorophenol or triclocarban, are high in concentration (Figure 5 and Table S9). Comparably high concentrations of fatty acids were observed in both freshwater sediment sites.

**TABLE 2:** Final method in each ionization mode

Parameter	ESI^−^	ESI^+^
Gradient (%B/min)	1.0	
Additive type	5mM AmFo	
Water (%)	0.0	5.0
Make‐up solvent flow rate (ml/min)	0.1	
Make‐up solvent additive	10mM FA	
Capillary voltage (kV)	1.5	3.0
Cone voltage (V)	30	

## 4 CONCLUDING REMARKS

A mixture of selected organic contaminants, an initial injection solvent screening, and two D‐optimal designs were used to improve UHPSFC‐ESI‐MS peak capacity and sensitivity prior to a nontarget screening of sediment samples. The highest peak capacities were obtained with the shallowest gradient slope (1.0%B/min) and AmFo as a modifier additive. With respect to ionization efficiency, the second D‐optimal design indicated that a lower additive concentration (here, 5mM) has a positive effect on the signal response, likely because ion suppression effects are reduced. Water in the modifier (5%) and a capillary voltage of 3.0kV were beneficial in ESI^+^. Conversely, a capillary voltage of 1.5kV and the absence of water improved the response in ESI^−^. A low make‐up solvent flow rate (0.1ml/min) increased the response in both ionization modes likely because of the reduced post‐column dilution; however, this effect was comparatively small and non‐significant. A better sensitivity was achieved with the optimized conditions compared to a design center point based on a literature survey. Results indicate that this method is suboptimal to analyse small acids due to the apparent pH of the modifier when no water is added. While this was deemed acceptable here, a different set of standards may be better suited for the optimization in case this class of compounds is relevant.

Finally, a set of sediment samples from Copenhagen were analyzed, detecting 35 of 50 of the selected organic contaminants, which shows the applicability of UHPSFC‐ESI‐MS for the analysis of solid environmental matrices.

## CONFLICT OF INTEREST

The authors declare that they have no conflict of interest.

## Supporting information

Supporting Information

## Data Availability

The data that support the findings of this study are available from the corresponding author upon reasonable request.
