# Supplementary material for: Ultra‐high‐performance supercritical fluid chromatography‐mass spectrometry for the analysis of organic contaminants in sediments
Source: J Sep Sci. 2022 Nov 10;46(1):2200668. doi: 10.1002/jssc.202200668 (PMC10099621; doi:10.1002/jssc.202200668)
Supplement: Supplementary file 1 — Supporting Information [file JSSC-46-0-s001.docx]

**SUPPLEMENTARY INFORMATION**

**Content**

Table S1. Primary standard solutions and nominal concentrations.

Table S2. List of standards, including suppliers.

Table S3. Conditions for injection solvent experiment.

Section S1. Model equations for the two D-optimal designs.

Table S4. Design space (*viz*. experiments that were run) and responses of *Design I*.

Figure S1. Gradient programme in *Design I*.

Table S5. Design space and experimental matrix of *Design II*.

Table S6. Conditions for quality control (QC) runs in the sequences of *Design I* and *II*.

Table S7. Normalised peak height (to the maximum across all tested injection solvents) for each individual compound in ESI^-^ and ESI^+^.

Figure S2. Blank injection of different injection solvents or mixtures.

Figure S3. Coefficients of *Design I* with peak capacity as the response for negative (ESI^-^) and positive ionisation (ESI+).

Table S8. Validation experiments of *Design I*.

Figure S4. Prediction plots with peak capacities of the five validation experiments of *Design I*.

Figure S5. Prediction plots of *Design II*.

Table S9. Concentrations [ngg^-1^] in sediment samples.

**Table S1.** Primary standard solutions and nominal concentrations in each solvent (acetone, ethanol:methanol [1:1] and methanol). *Italic:* Compounds that were in the mixtures but do not ionise in ESI^+^ or ESI^-^ and were therefore disregarded.

| **Compounds** | **Concentration [ppm]** |
| --- | --- |
| ***Mixture 1 (in acetone)*** |  |
| 1-adamantanecarboxylic acid, 1-hydroxy-2-naphthoic acid, 1-naphthoic acid, 1-pyrenecarboxylic acid, 2-carboxycinnamic acid, 2-naphthoic acid, 4-butylbenzoic acid, acetophenone, anisole, benzonitrile, bisphenol A, cyclohexanecarboxylic acid, cyclohexanepentanoic acid, dicyclohexylacetic acid, diphenic acid, estrone, lauric acid, linoleic acid, linolenic acid, oleic acid, palmitoleic acid, perfluorooctanoic acid (PFOA), perfluorooctanesulfonic acid (PFOS), phthalic acid, progesterone, salicylic acid, testosterone, trans-chalcone, *3,3',5,5'-tetrabromo-1,1'-biphenyl, 4,4'-DDT, 4,4'-dibromobiphenyl, benzo(a)pyrene, bu­tylbenzene, DFTPP, ethylbenzene, fluoranthene* | 38.4±2.4 |
| ***Mixture 2 (in ethanol/methanol [1:1])*** |  |
| 1H-benzo(g)indole, 2-mercaptobenzothiazole, 2-phenylphenol, acridine, amitrip­tyline, carbamazepine, carbazole, cypermethrin, imazalil, imidacloprid, isoquinoline, N,N-diethylacetamide, N-ethylcarbazole, pentachlorophenol, pirimicarb, pyrocatechol, quinoxaline, tebuconazole, triclocarban, triclosan | 38.7±2.6 |
| ***Mixture 3 (in methanol)*** |  |
| 1,7-dihydroxynaphthalene, 9,10-anthraquinone, 9-hydroxyphenanthrene, dibenzofuran | 28.3±0.8 |

Table S2. List of standards, including monoisotopic mass, compound group, supplier and identifier. *Italic*: Compounds that were in the mixtures but do not ionise in ESI^+^ or ESI^-^ and were therefore disregarded. ^a^) Qualifier ion not observed in MS SCAN, but second MS channel, that was ramped with a cone voltage 10-50 V; retention times in final UHPSFC-MS method.

| **Compound** | **Functional use** | **Supplier** | **InChiKey** | **Monoisotopic mass [Da]** | **ESI mode** | **Quantifier 🡪 Qualifier ion** | **Retention time [min]** |
| --- | --- | --- | --- | --- | --- | --- | --- |
| 1,7-Dihydroxynaphthalene | Colorant | Acros Organics | ZUVBIBLYOCVYJU-HFFFAOYSA-N | 160.05240 | NEG | 159.0446 🡪 158.0380 | 14.09 |
| 1-Adamantanecarboxylic acid | Industrial precursor and intermediate | Aldrich | JIMXXGFJRDUSRO-UHFFFAOYSA-N | 180.11500 | NEG | 179.1072 | 4.24 |
| 1H-Benzo(g)indole | Industrial precursor and intermediate | Sigma-Aldrich | HIYWOHBEPVGIQN-UHFFFAOYSA-N | 167.07350 | NEG | 166.069 | 4.10 |
| 1-Hydroxy-2-naphthoic acid | Metabolite, industrial precursor and intermediate | Aldrich | SJJCQDRGABAVBB-UHFFFAOYSA-N | 188.04730 | NEG | 187.0380 🡪143.0510 | 19.77 |
| 1-Naphthoic acid | Industrial precursor and intermediate | Alfa Aesar | LNETULKMXZVUST-UHFFFAOYSA-N | 172.05240 | NEG | 171.0446 🡪 127.0560 | 7.12 |
| 1-Pyrenecarboxylic acid | Metabolite, industrial precursor and intermediate | Sigma-Aldrich | HYISVWRHTUCNCS-UHFFFAOYSA-N | 246.06808 | NEG | 245.061 🡪 201.0697 | 13.17 |
| 2-Carboxycinnamic acid | Metabolite | Alfa Aesar | SCWPNMHQRGNQHH-AATRIKPKSA-N | 192.04230 | NEG | - | - |
| 2-Mercaptobenzothiazole | Catalyst | Aldrich | YXIWHUQXZSMYRE-UHFFFAOYSA-N | 166.98630 | NEG  POS | 165.9785 🡪 134.005  167.9942 | 12.25  11.29 |
| 2-Naphthoic acid | Metabolite, industrial precursor and intermediate | Aldrich | UOBYKYZJUGYBDK-UHFFFAOYSA-N | 172.05240 | NEG | 171.0446 🡪 127.0560 | 8.06 |
| 2-Phenylphenol | Antimicrobial | Fluka | LLEMOWNGBBNAJR-UHFFFAOYSA-N | 170.07320 | NEG | 169.0653 | 1.75 |
| *3,3',5,5'-tetrabromo-1,1'-biphenyl* | *Flame retardant* | *Aldrich* | *FXJXZYWFJAXIJX-UHFFFAOYSA-N* | *465.72030* | *-* | *-* | *-* |
| *4,4'-DDT* | *Insecticide* | *Fluka* | *YVGGHNCTFXOJCH-UHFFFAOYSA-N* | *351.91470* | *-* | *-* | *-* |
| *4,4'-Dibromobiphenyl* | *Flame retardant* | *Aldrich* | *HQJQYILBCQPYBI-UHFFFAOYSA-N* | *309.89930* | *-* | *-* | *-* |
| 4-Butylbenzoic acid | Industrial precursor and intermediate | Aldrich | JFKUBRAOUZEZSL-UHFFFAOYSA-N | 178.09940 | NEG | 177.094 🡪 133.102 | 5.25 |
| 9,10-Anthraquinone | Colorant | Sigma-Aldrich | RZVHIXYEVGDQDX-UHFFFAOYSA-N | 208.05240 | POS | 209.06 | - |
| 9-Hydroxyphenanthrene | Metabolite | Sigma-Aldrich | DZKIUEHLEXLYKM-UHFFFAOYSA-N | 194.07320 | NEG | 193.0653 🡪 165.0702 | 8.66 |
| Acetophenone | Fragrance | Sigma-Aldrich | KWOLFJPFCHCOCG-UHFFFAOYSA-N | 120.05750 | POS | 121.0653 | - |
| Acridine | Colorant | Alfa Aesar | DZBUGLKDJFMEHC-UHFFFAOYSA-N | 179.07350 | POS | 180.0813 | 1.78 |
| Amitriptyline | Pharmaceutical | Sigma | KFYRPLNVJVHZGT-UHFFFAOYSA-N | 313.15973 | POS | 278.1909 🡪 233.132 | 7.99 |
| Anisole | Fragrance | Fluka | RDOXTESZEPMUJZ-UHFFFAOYSA-N | 108.05750 | POS | 109.064 | 6.58 |
| *Benzo(a)pyrene* | PAH | *Sigma-Aldrich* | *FMMWHPNWAFZXNH-UHFFFAOYSA-N* | *252.09390* | *-* | *-* | *-* |
| Benzonitrile | Fragrance, industrial precursor and intermediate | Fluka | JFDZBHWFFUWGJE-UHFFFAOYSA-N | 103.04220 | POS | 104.050 | 0.66 |
| Bisphenol A | Crosslinker | Sigma-Aldrich | IISBACLAFKSPIT-UHFFFAOYSA-N | 228.11500 | NEG | 227.1072 🡪 212.084 ^a^ | 12.45 |
| *Butylbenzene* | Organic solvent | *Aldrich* | *OCKPCBLVNKHBMX-UHFFFAOYSA-N* | *134.10960* | *-* | *-* | *-* |
| Carbamazepine | Pharmaceutical | Sigma | FFGPTBGBLSHEPO-UHFFFAOYSA-N | 236.09500 | POS | 237.1028 🡪 194.097 | 8.11 |
| Carbazole | Colorant | Fluka | UJOBWOGCFQCDNV-UHFFFAOYSA-N | 167.07350 | NEG | 166.069 | 5.61 |
| Cyclohexanecarboxylic acid | Fragrance | Sigma-Aldrich | NZNMSOFKMUBTKW-UHFFFAOYSA-N | 128.08370 | NEG | 127.0759 | - |
| Cyclohexanepentanoic acid | Organic solvent | Aldrich | YMUHUYBRWUUAJF-UHFFFAOYSA-N | 184.14630 | NEG | 183.1385 | 3.14 |
| Cypermethrin | Insecticide |  | KAATUXNTWXVJKI-UHFFFAOYSA-N | 415.07420 | POS | 416.0820 | 0.67 |
| *DFTPP* | *System suitability test GC* | *Supelco* | *OYNXPGGNQMSMTR-UHFFFAOYSA-N* | *441.99690* | *-* | *-* | *-* |
| Dibenzofuran | Fragrance, industrial precursor | Fluka | TXCDCPKCNAJMEE-UHFFFAOYSA-N | 168.05750 | POS | 169.0653 | 1.79 |
| Dicyclohexylacetic acid | Industrial precursor and intermediate | Aldrich | PGGMEZOUAPIYOY-UHFFFAOYSA-N | 224.17760 | NEG | 223.1698 | 3.88 |
| Diphenic acid | Industrial precursor and intermediate, colorant | Aldrich | GWZCCUDJHOGOSO-UHFFFAOYSA-N | 242.05790 | NEG | 241.0501 🡪 197.059 | 23.25 |
| Estrone | Steroid | Sigma-Aldrich | DNXHEGUUPJUMQT-CBZIJGRNSA-N | 270.16200 | NEG  POS | 269.1542 🡪 145.063^a^  271.1698 🡪 253.159 | 7.68  7.71 |
| *Ethylbenzene* | *Industrial precursor and intermediate* | *Fluka* | *YNQLUTRBYVCPMQ-UHFFFAOYSA-N* | *106.07830* | *-* | *-* | *-* |
| *Fluoranthene* | *PAH* | *Sigma-Aldrich* | *GVEPBJHOBDJJJI-UHFFFAOYSA-N* | *202.07820* | *-* | *-* | *-* |
| Imazalil | Fungicide | Sigma-Aldrich | PZBPKYOVPCNPJY-UHFFFAOYSA-N | 296.04832 | POS | 297.0561 🡪 299.055 | 3.86 |
| Imidacloprid | Insecticide | Sigma-Aldrich | YWTYJOPNNQFBPC-UHFFFAOYSA-N | 255.05230 | NEG  POS | 254.0445 🡪 256.0445  256.0601 🡪 258.057 | 10.46  11.08 |
| Isoquinoline | Flavorant, industrial precursor and intermediate | Sigma-Aldrich | AWJUIBRHMBBTKR-UHFFFAOYSA-N | 129.05780 | POS | 130.066 | 1.04 |
| Lauric acid | Fatty acid | SAFC | POULHZVOKOAJMA-UHFFFAOYSA-N | 200.17760 | NEG | 199.1698 | 2.58 |
| Linoleic acid | Fatty acid | Sigma | OYHQOLUKZRVURQ-HZJYTTRNSA-N | 280.24020 | NEG | 279.2324 | 3.57 |
| Linolenic acid | Fatty acid | Sigma | DTOSIQBPPRVQHS-PDBXOOCHSA-N | 278.22460 | NEG | 277.2168 | 3.82 |
| N,N-Diethylacetamide | Organic solvent | Aldrich | AJFDBNQQDYLMJN-UHFFFAOYSA-N | 115.09970 | NEG | 116.1075 | 0.97 |
| N-Ethylcarbazole | Industrial precursor and intermediate | Sigma-Aldrich | PLAZXGNBGZYJSA-UHFFFAOYSA-N | 195.10480 | POS | 196.1126 🡪 167.076 | 0.58 |
| Oleic acid | Fatty acid | Sigma-Aldrich | ZQPPMHVWECSIRJ-KTKRTIGZSA-N | 282.25590 | NEG | 281.2481 | 3.33 |
| Palmitoleic acid | Fatty acid | Sigma | SECPZKHBENQXJG-FPLPWBNLSA-N | 254.22460 | NEG | 253.2168 | 3.14 |
| Pentachlorophenol | Antimicrobial | Supelco | IZUPBVBPLAPZRR-UHFFFAOYSA-N | 263.84700 | NEG | 264.8366 🡪 266.833 | 7.98 |
| Perfluorooctanesulfonic acid | Flame retardant, emulsion stabilizer | Aldrich | WFRUBUQWJYMMRQ-UHFFFAOYSA-M | 537.89340 | NEG | 498.9301 🡪 79.957^a^ | 15.60 |
| Perfluorooctanoic acid | Flame retardant, emulsion stabilizer | Aldrich | SNGREZUHAYWORS-UHFFFAOYSA-N | 413.97370 | NEG | 368.9775 🡪 412.9656 | 13.18 |
| Phthalic acid | Colorant, preservative | Fluka | XNGIFLGASWRNHJ-UHFFFAOYSA-N | 166.02660 | NEG | 165.0188 | 24.51 |
| Pirimicarb | Insecticide | Sigma-Aldrich | YFGYUFNIOHWBOB-UHFFFAOYSA-N | 238.14298 | POS | 239.1508 🡪 182.130 | 1.01 |
| Progesterone | Steroid | Fluka | RJKFOVLPORLFTN-LEKSSAKUSA-N | 314.22460 | POS | 315.2324 🡪 97.064 | 1.92 |
| Pyrocatechol | Industrial precursor and intermediate | Fluka | YCIMNLLNPGFGHC-UHFFFAOYSA-N | 110.03680 | NEG | 109.0290 | 9.15 |
| Quinoxaline | Industrial precursor and intermediate | Sigma-Aldrich | XSCHRSMBECNVNS-UHFFFAOYSA-N | 130.05310 | POS | 131.0609 | 0.66 |
| Salicylic acid | Preservative | Sigma-Aldrich | YGSDEFSMJLZEOE-UHFFFAOYSA-N | 138.03170 | NEG | 137.0244 | - |
| Tebuconazole | Fungicide | Sigma-Aldrich | PXMNMQRDXWABCY-UHFFFAOYSA-N | 307.14514 | POS | 308.1530 🡪 310.151 | 4.88 |
| Testosterone | Steroid | Fluka | MUMGGOZAMZWBJJ-DYKIIFRCSA-N | 288.20890 | POS | 289.2168 🡪 109.064 | 6.55 |
| Trans-chalcone | Pharmaceutical | Aldrich | DQFBYFPFKXHELB-VAWYXSNFSA-N | 208.08880 | POS | 209.0966 🡪 131.049 | 0.71 |
| Triclocarban | Antimicrobial | Sigma-Aldrich | ICUTUKXCWQYESQ-UHFFFAOYSA-N | 313.97800 | NEG  POS | 159.9758 🡪 312.9708  314.9859 🡪 316.9859 | 12.38  10.76 |
| Triclosan | Antimicrobial | Fluka | XEFQLINVKFYRCS-UHFFFAOYSA-N | 287.95120 | NEG | 286.9433 🡪 288.9373 | 3.61 |
|  |  |  |  |  |  |  |  |

Table S3. Conditions for injection solvent experiments.

| **Parameters (Chromatography)** | | **Parameters (Mass spectrometry)** | |
| --- | --- | --- | --- |
| Column | Torus DIOL (3×100 mm, 1.7 µm) | Capillary voltage | 2.25 kV |
| Mobile phase | CO_2_ [A]: 0.1% FA in MeOH [B] | Cone voltage | 25 V |
| Flow rate | 1.5 mL min^-1^ | Source temperature | 135 °C |
| Injection volume | 3 µL | Desolvation temperature | 525 °C |
| Column temperature | 40 °C | Cone gas flow | 100 L hr^-1^ |
| ABPR | 140 bar | Desolvation gas flow | 1000 L hr^-1^ |
| Make-up solvent | 0.1% FA in MeOH | Scan rate | 0.2 scan s^-1^ |
| Make-up solvent flow rate | 0.1 mL min^-1^ | Mass range | 50 – 1,200 Da |
| Gradient programme | Initial [A:B]: 99.5:0.5  0-3 min: 99.5:0.5  3-13 min: 85:15  13-18 min: 70:30  18-21 min: 70:30  21-21.1 min: 99.5:0.5  21.1-24 min: 99.5:0.5 | Scan mode | Centroid |

**Section S1.** Models – *Design I* and *II*

*Design I* consisted of three factors and eight coefficients. The model was as follows:

$$y=b_{0}+b_{FA}FA+b_{AmAc}AmAc+b_{AmFo}AmFo+b_{w}w+b_{g}g+b_{gw}gw+b_{g^{2}}g^{2}$$

where $y$ is the response, $b_{0}$ the constant term for the intercept, *b_x_X* the particular linear ($b_{FA}FA$, $b_{AmAc}AmAc$, $b_{AmFo}AmFo$, $b_{w}w$ and $b_{g}g$), interaction ($b_{gw}gw$) or quadratic term ($b_{g^{2}}g^{2}$).

*Design II* consisted of six factors and 27 coefficients. The model was as follows:

$$y=b_{0}+b_{\mathrm{CapV}}x_{\mathrm{CapV}}+\ldots+b_{\mathrm{Add}}x_{\mathrm{Add}}+b_{\mathrm{CapV}^{2}}x_{\mathrm{CapV}^{2}}+\ldots+b_{\mathrm{Add}^{2}}x_{\mathrm{Add}^{2}}+b_{\mathrm{CapVConeV}}x_{\mathrm{CapV}}x_{\mathrm{ConeV}}+{\ldots+b}_{\mathrm{CapVAdd}}x_{\mathrm{CapV}}x_{\mathrm{Add}}+b_{\mathrm{ConeVmuF}}x_{\mathrm{ConeV}}x_{\mathrm{muF}}+{\ldots+b}_{\mathrm{ConeVAdd}}x_{\mathrm{ConeV}}x_{\mathrm{Add}}+b_{\mathrm{muFmuPH}}x_{\mathrm{muF}}x_{\mathrm{muPH}}+{\ldots+b}_{\mathrm{muFAdd}}x_{\mathrm{muF}}x_{\mathrm{Add}}+b_{\mathrm{muPHw}}x_{\mathrm{muPH}}x_{w}{+b}_{\mathrm{muPHAdd}}x_{\mathrm{muPH}}x_{\mathrm{Add}}{+b}_{\mathrm{wAdd}}x_{w}x_{\mathrm{Add}}$$

where $y$ is the response, $b_{0}$ the constant term for the intercept, *b_x_X* the particular linear ($b_{CapV}CapV$, $b_{ConeV}ConeV$, $b_{muF}muF$, $b_{muPH}muPH$, $b_{w}w$ and $b_{Add}Add$), binary interaction ($b_{CapVConeV}CapV\cdot ConeV$, $b_{CapVmuF}CapV\cdot muF$, $b_{CapVmuPH}CapV\cdot muPH$, $b_{CapVw}CapV\cdot w$, $b_{CapVAdd}CapV\cdot Add$, $b_{ConeVmuF}ConeV\cdot muF$, $b_{ConeVmuPH}ConeV\cdot muPH$, $b_{ConeVw}ConeV\cdot w$, $b_{ConeVAdd}Cone\cdot Add$, $b_{muFmuPH}muF\cdot muPH$, $b_{muFw}muF\cdot w$, $b_{muFAdd}muF\cdot Add$, $b_{muPHw}muPH\cdot w$, $b_{muPHAdd}muPH\cdot Add$, $b_{wAdd}w\cdot Add$) or quadratic term ($b_{CapV^{2}}CapV^{2}$, $b_{ConeV^{2}}ConeV^{2}$, $b_{muF^{2}}muF^{2}$, $b_{w^{2}}w^{2}$, $b_{Add^{2}}{Add}^{2}$).

Table S4. Experiment matrix (*viz*. experiments that were run) and responses of *Design I*. For explanations of the abbreviations, please refer to the main text. The use of additive was coded with +1 in the corresponding design column. In practice, this entails that the model coefficients can be interpreted as the effect of the additive compared to pure methanol as modifier (which was coded with 0).

| **Exp No.** | **Matrix of candidate points** | | | | | **Response** | | | | | | | |
| --- | --- | --- | --- | --- | --- | --- | --- | --- | --- | --- | --- | --- | --- |
|  | **FA** | **AmAc** | **AmFo** | **w** | **g** | **ESI^-^** | | | | **ESI^+^** | | | |
|  |  |  |  |  |  | **Peak capacity** | **# of missing compounds** | **Average area** | **A_sc_** | **Peak capacity** | **# of missing compounds** | **Average area** | **A_sc_** |
| **1** | +1 | 0 | 0 | -1 | 0 | 122 | 0 | 18447 | 0.46 | 122 | 3 | 47013 | 0.17 |
| **2** | 0 | 0 | 0 | +1 | 0 | 175 | 2 | 5951 | 0.06 | 113 | 3 | 37771 | 0.17 |
| **3** | 0 | 0 | 0 | -1 | -1 | 217 | 0 | 22967 | 0.76 | 142 | 3 | 70076 | 0.31 |
| **4** | 0 | 0 | 0 | -1 | +1 | 104 | 0 | 25147 | 0.83 | 61 | 3 | 55894 | 0.23 |
| **5** | +1 | 0 | 0 | +1 | -1 | 353 | 7 | 6636 | 0.07 | 183 | 3 | 98382 | 0.69 |
| **6** | +1 | 0 | 0 | +1 | +1 | 121 | 8 | 6023 | 0.07 | 78 | 3 | 143948 | 0.98 |
| **7** | 0 | 0 | +1 | -1 | 0 | 160 | 3 | 14055 | 0.40 | 141 | 3 | 32873 | 0.18 |
| **8** | 0 | 0 | +1 | +1 | +1 | 131 | 2 | 4047 | 0.07 | 103 | 3 | 25097 | 0.12 |
| **9** | 0 | 0 | +1 | +1 | -1 | 378 | 2 | 5280 | 0.11 | 324 | 3 | 38688 | 0.25 |
| **10** | 0 | +1 | 0 | +1 | 0 | 186 | 1 | 4612 | 0.25 | 138 | 3 | 31351 | 0.19 |
| **11** | 0 | +1 | 0 | -1 | +1 | 138 | 1 | 6145 | 0.27 | 94 | 3 | 14683 | 0.03 |
| **12** | 0 | +1 | 0 | -1 | -1 | 294 | 1 | 7833 | 0.38 | 327 | 3 | 15691 | 0.04 |


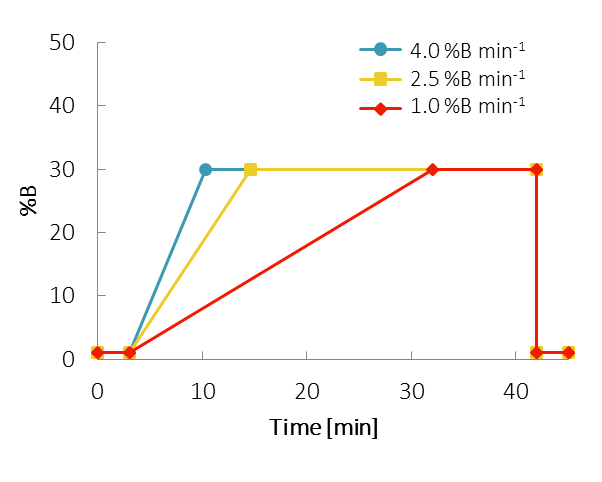


Figure S1. Gradient programme in *Design I.*

Table S5. Design space and experimental matrix of *Design II*. For explanations of the abbreviations, please refer to the main text. Experimental number reflects the index in the original search space and bears no connection to the actual number of experiments that were run.

|  | **Matrix of candidate points** | | | | | | **Experimental matrix** | | | | | | **Results ESI^-^** | | **Results ESI^+^** | | |
| --- | --- | --- | --- | --- | --- | --- | --- | --- | --- | --- | --- | --- | --- | --- | --- | --- | --- |
| **Exp No.** | **CapV** | **ConeV** | **muF** | **muPH** | **[w]** | **[Add]** | **CapV [kV]** | **ConeV [V]** | **muF**  **[mL min^-1^]** | **muPH [10 mM]** | **[w] [%]** | **[Add] [mM]** | **Peak capacity** | **A_sc_** | **Peak capacity** | | **A_sc_** |
| **3** | -1 | +1 | -1 | +1 | +1 | +1 | 1.5 | 30 | 0.1 | NH_3_ | 5 | 20 | 368.0 | 0.05 | 299.9 | 0.16 | |
| **15** | -1 | +1 | -1 | -1 | 0 | +1 | 1.5 | 30 | 0.1 | FA | 2.5 | 20 | 329.7 | 0.13 | 284.8 | 0.14 | |
| **16 ^c^** | -1 | +1 | -1 | -1 | -1 | -1 | 1.5 | 30 | 0.1 | FA | 0 | 5 | 288.0  289.7 | 0.69  0.80 | 274.6  272.1 | 0.12  0.29 | |
| **25 ^a^** | -1 | +1 | 0 | +1 | -1 | -1 | 1.5 | 30 | 0.15 | NH_3_ | 0 | **5** | 321.4  303.9 | 0.43  0.52 | 260.8  274.6 | 0.15  0.05 | |
| **29 ^a^** | -1 | +1 | 0 | -1 | +1 | 0 | 1.5 | 30 | 0.15 | FA | 5 | 12.5 | 344.7  334.2 | 0.17  0.18 | 273.8  274.3 | 0.36  0.35 | |
| **45 ^a^** | -1 | +1 | +1 | +1 | -1 | +1 | 1.5 | 30 | 0.2 | NH_3_ | 0 | 20 | 314.5  307.9 | 0.28  0.22 | 283.7  290.6 | 0.14  0.11 | |
| **49 ^c^** | -1 | +1 | +1 | -1 | 0 | -1 | 1.5 | 30 | 0.2 | FA | 2.5 | 5 | 340.4  331.5 | 0.42  0.47 | 267.2  271.1 | 0.30  0.50 | |
| **90 ^a^** | -1 | 0 | 0 | -1 | -1 | +1 | 1.5 | 25 | 0.15 | FA | 0 | 20 | 301.8 | 0.36 | 279.9  292.2 | 0.13  0.10 | |
| **91** | -1 | 0 | +1 | +1 | +1 | -1 | 1.5 | 25 | 0.2 | NH_3_ | 5 | 5 | 338.7 | 0.37 | 282.0 | 0.29 | |
| **113 ^a^** | -1 | -1 | -1 | +1 | 0 | 0 | 1.5 | 20 | 0.1 | NH_3_ | 2.5 | 12.5 | 327.4  331.1 | 0.16  0.18 | 283.4  281.3 | 0.19  0.18 | |
| **117** | -1 | -1 | -1 | +1 | -1 | +1 | 1.5 | 20 | 0.1 | NH_3_ | 0 | 20 | 319.1 | 0.33 | 278.3 | 0.17 | |
| **118 ^c^** | -1 | -1 | -1 | -1 | +1 | -1 | 1.5 | 20 | 0.1 | FA | 5 | 5 | 350.9  333.3 | 0.45  0.49 | 267.6 | 0.52 | |
| **150** | -1 | -1 | +1 | +1 | 0 | +1 | 1.5 | 20 | 0.2 | NH_3_ | 2.5 | 20 | 313.5 | 0.10 | 295.8 | 0.12 | |
| **156** | -1 | -1 | +1 | -1 | +1 | +1 | 1.5 | 20 | 0.2 | FA | 5 | 20 | 338.3 | 0.06 | 284.1 | 0.20 | |
| **160 ^c^** | -1 | -1 | +1 | -1 | -1 | -1 | 1.5 | 20 | 0.2 | FA | 0 | 5 | 310.3  296.4  296.1 | 0.69  0.73  0.72 | 266.2  277.0 | 0.17  0.28 | |
| **166 ^a,c^** | 0 | +1 | -1 | +1 | 0 | -1 | 2.25 | 30 | 0.1 | NH_3_ | 2.5 | 5 | 312.5  312.5  314.4 | 0.51  0.51  0.64 | 291.6  286.1  286.3  293.0 | 0.28  0.32  0.35  0.46 | |
| **210** | 0 | +1 | +1 | -1 | +1 | +1 | 2.25 | 30 | 0.2 | FA | 5 | 20 | 318.7 | 0.08 | 288.8 | 0.12 | |
| **260** | 0 | 0 | +1 | +1 | -1 | 0 | 2.25 | 25 | 0.2 | NH_3_ | 0 | 12.5 | 291.6 | 0.18 | 280.8 | 0.10 | |
| **291 ^a^** | 0 | -1 | 0 | +1 | +1 | +1 | 2.25 | 20 | 0.15 | NH_3_ | 5 | 20 | 349.3  341.5 | 0.12  0.10 | 284.7  285.3 | 0.10  0.10 | |
| **305 ^a,c^** | 0 | -1 | 0 | -1 | -1 | 0 | 2.25 | 20 | 0.15 | FA | 0 | 12.5 | 290.6  297.2 | 0.33  0.37 | 279.4  286.0  269.5 | 0.15  0.11  0.15 | |
| **332** | +1 | +1 | -1 | +1 | -1 | 0 | 3 | 30 | 0.1 | NH_3_ | 0 | 12.5 | 309.0 | 0.18 | 285.8 | 0.18 | |
| **334** | +1 | +1 | -1 | -1 | +1 | -1 | 3 | 30 | 0.1 | FA | 5 | 5 | 344.0 | 0.40 | 272.8 | 0.89 | |
| **342** | +1 | +1 | -1 | -1 | -1 | +1 | 3 | 30 | 0.1 | FA | 0 | 20 | 316.0 | 0.29 | 266.5 | 0.17 | |
| **348 ^a^** | +1 | +1 | 0 | +1 | 0 | +1 | 3 | 30 | 0.15 | NH_3_ | 2.5 | 20 | 316.4  319.4 | 0.26  0.19 | 282.8  279.8 | 0.13  0.13 | |
| **361** | +1 | +1 | +1 | +1 | +1 | -1 | 3 | 30 | 0.2 | NH_3_ | 5 | 5 | 336.7 | 0.43 | 281.1 | 0.27 | |
| **372** | +1 | +1 | +1 | -1 | +1 | +1 | 3 | 30 | 0.2 | FA | 5 | 20 | 331.9 | 0.06 | 289.5 | 0.08 | |
| **376** | +1 | +1 | +1 | -1 | -1 | -1 | 3 | 30 | 0.2 | FA | 0 | 5 | 312.6 | 0.36 | 267.6 | 0.11 | |
| **409** | +1 | 0 | 0 | -1 | 0 | -1 | 3 | 25 | 0.15 | FA | 2.5 | 5 | 328.1 | 0.32 | 278.0 | 0.21 | |
| **417** | +1 | 0 | +1 | +1 | +1 | +1 | 3 | 25 | 0.2 | NH_3_ | 5 | 20 | 343.9 | 0.10 | 291.0 | 0.10 | |
| **433 ^c^** | +1 | -1 | -1 | +1 | +1 | -1 | 3 | 20 | 0.1 | NH_3_ | 5 | 5 | 341.1  328.5  333.6 | 0.42  0.41  0.39 | 273.8  273.4  269.4 | 0.92  0.70  0.31 | |
| **441** | +1 | -1 | -1 | +1 | -1 | +1 | 3 | 20 | 0.1 | NH_3_ | 0 | 20 | 304.9 | 0.32 | 294.9 | 0.15 | |
| **444** | +1 | -1 | -1 | -1 | +1 | +1 | 3 | 20 | 0.1 | FA | 5 | 20 | 337.4 | 0.07 | 288.7 | 0.24 | |
| **448** | +1 | -1 | -1 | -1 | -1 | -1 | 3 | 20 | 0.1 | FA | 0 | 5 | 306.3 | 0.36 | 276.8 | 0.15 | |
| **475** | +1 | -1 | +1 | +1 | -1 | -1 | 3 | 20 | 0.2 | NH_3_ | 0 | 5 | 299.6 | 0.43 | 263.1 | 0.03 | |
| **478 ^a,c^** | +1 | -1 | +1 | -1 | +1 | -1 | 3 | 20 | 0.2 | FA | 5 | 5 | 343.4  332.0  339.5 | 0.44  0.49  0.24 | 276.0  276.0 | 0.69  0.26 | |
| **486** | +1 | -1 | +1 | -1 | -1 | +1 | 3 | 20 | 0.2 | FA | 0 | 20 | 327.8 | 0.18 | 283.5 | 0.13 | |
| **124 ^b^** | -1 | -1 | -1 | -1 | -1 | -1 | 1.5 | 20 | 0.1 | FA | 0 | 5 | 294.4  297.5 | 0.72  0.64 | - | - | |
| **388 ^b^** | +1 | 0 | -1 | -1 | +1 | -1 | 3 | 25 | 0.1 | FA | 5 | 5 | - | - | 272.8  266.9 | 0.54  0.74 | |
| **248 ^b,d^** | 0 | 0 | 0 | -1 | 0 | 0 | 2.25 | 25 | 0.15 | FA | 2.5 | 12.5 | - | - | - | - | |

^a)^ These experiments were run in duplicates;

^b)^ Experiments were added during validation;

^c)^ Experiments were repeated during validation;

^d)^ Centre point across all conditions except for muPH (10 mM FA).

Table S6. Conditions for quality controls (QC) or facilitator samples in the sequences of *Design I* and *II*.

| **Parameters (Chromatography)** | | **Parameters (Mass spectrometry)** | |
| --- | --- | --- | --- |
| Column | Torus DIOL (3×100 mm, 1.7 µm) | Capillary voltage | 1.5 kV |
| Mobile phase | CO_2_ [A]: MeOH [B] | Cone voltage | 30 V |
| Flow rate | 1.5 mL min^-1^ | Source temperature | 120 °C |
| Injection volume | 3 µL | Desolvation temperature | 500 °C |
| Column temperature | 40 °C | Cone gas flow | 100 L hr^-1^ |
| ABPR | 140 bar | Desolvation gas flow | 1000 L hr^-1^ |
| Make-up solvent | 0.1% FA in MeOH | Scan rate | 0.2 scan s^-1^ |
| Make-up solvent flow rate | 0.1 mL min^-1^ | Mass range | 50 – 1,200 Da |
| Gradient programme | Initial [A:B]: 99:1  0-3 min: 99:1  3-8.5 min: 70:30  8.5-20 min: 70:30  20-20.5 min: 99:1  20.5-25 min: 99:1 | Scan type | Centroid |

Table S7. Normalised peak height (to the maximum across all tested injection solvents) for each individual compound in ESI^-^ and ESI^+^. Median and relative standard deviation (RSD) in [%] were calculated for all compounds.

| **Compound** | **t_R_ [min] ^a^** | **DCM: acetone** | **THF** | **Hexane** | **MTBE** | **DCM:acetone:hexane** |
| --- | --- | --- | --- | --- | --- | --- |
| *ESI^-^(n=29)* |  |  |  |  |  |  |
| 1,7-Dihydroxy­naphthalene | 11.98 | **1.00** | 0.92 | 0.02 | 0.69 | 0.55 |
| 1-Hydroxy-2-naphthoic acid | 11.04 | 0.93 | **1.00** | 0.37 | 0.89 | 0.77 |
| 1-Naphthoic acid | 6.37 | 0.92 | **1.00** | 0.73 | 0.84 | 0.76 |
| 1-Pyrenecarboxylic acid | 9.42 | 0.98 | **1.00** | 0.64 | 0.78 | 0.70 |
| 2-Carboxycinnamic acid | 11.98 | 0.97 | **1.00** | 0.06 | 0.83 | 0.65 |
| 2-Mercaptobenzothiazole | 7.61 | **1.00** | 0.65 | 0.25 | 0.10 | 0.51 |
| 2-Naphthoic acid | 6.69 | 0.95 | **1.00** | 0.65 | 0.80 | 0.76 |
| 2-Phenylphenol | 1.80 | **1.00** | 0.92 | 0.78 | 0.75 | 0.67 |
| 4-Butylbenzoic acid | 5.29 | 0.89 | **1.00** | 0.95 | 0.91 | 0.89 |
| 9-Hydroxyphenanthrene | 8.05 | **1.00** | 0.90 | 0.78 | 0.54 | 0.69 |
| Amitriptyline | 4.87 | **1.00** | 0.78 | 0.94 | 0.75 | 0.76 |
| Bisphenol a | 10.68 | **1.00** | 0.92 | 0.07 | 0.72 | 0.72 |
| Cyclohexanecarboxylic acid | 3.73 | **1.00** | 0.73 | 0.99 | 0.70 | 0.94 |
| Cyclohexanepentanoic acid | 4.28 | **1.00** | 0.56 | 0.80 | 0.66 | 0.76 |
| Dicyclohexylacetic acid | 4.87 | **1.00** | 0.74 | 0.81 | 0.73 | 0.74 |
| Diphenic acid | 14.55 | 0.39 | **1.00** | 0.02 | 0.44 | 0.23 |
| Estrone | 7.23 | **1.00** | 0.81 | 0.50 | 0.64 | 0.71 |
| Imidacloprid | 9.24 | **1.00** | 0.96 | 0.00 | 0.78 | 0.75 |
| Lauric acid | 3.50 | **1.00** | 0.60 | 0.96 | 0.68 | 0.77 |
| Linoleic acid | 4.59 | **1.00** | 0.61 | 0.77 | 0.82 | 0.77 |
| Linolenic acid | 4.70 | **1.00** | 0.60 | 0.81 | 0.62 | 0.77 |
| Oleic acid | 4.46 | 0.93 | 0.60 | 0.71 | **1.00** | 0.70 |
| Palmitoleic acid | 4.31 | **1.00** | 0.46 | 0.77 | 0.63 | 0.71 |
| Pentachlorophenol | 6.12 | **1.00** | 0.94 | 0.81 | 0.71 | 0.76 |
| Phthalic acid | 5.42 | 0.98 | 0.66 | 0.76 | 0.81 | **1.00** |
| Pyrocatechol | 8.00 | **1.00** | **1.00** | 0.10 | 0.81 | 0.73 |
| Salicylic acid | 8.86 | 0.80 | **1.00** | 0.10 | 0.94 | 0.74 |
| Triclocarban | 10.42 | **1.00** | 0.96 | 0.76 | 0.91 | 0.84 |
| Triclosan | 4.00 | **1.00** | 0.66 | 0.84 | 0.75 | 0.73 |
| *ESI^+^ (n=19)* |  |  |  |  |  |  |
| 1H-Benzo(g)indole | 5.67 | 0.98 | **1.00** | 0.33 | 0.92 | 0.91 |
| 9,10-Anthraquinone | 9.31 | 0.97 | **1.00** | 0.92 | 0.91 | 0.97 |
| Acridine | 2.43 | 0.91 | **1.00** | 0.92 | 0.93 | 0.81 |
| Amitriptyline | 4.87 | **1.00** | **1.00** | 0.79 | 0.80 | 0.93 |
| Carbamazepine | 7.63 | **1.00** | **1.00** | 0.85 | 0.89 | 0.93 |
| Carbazole | 0.68 | **1.00** | **1.00** | 0.36 | 0.73 | 0.88 |
| Estrone | 7.23 | 0.96 | 0.99 | **1.00** | 0.90 | 0.89 |
| Imazalil | 4.99 | 0.90 | 0.68 | 0.96 | **1.00** | 0.86 |
| Imidacloprid | 9.31 | 0.83 | 0.90 | 0.68 | **1.00** | 0.77 |
| Isoquinoline | 1.09 | 0.98 | **1.00** | 0.01 | 0.98 | 0.91 |
| N,N-diethylacetamide | 1.01 | 0.93 | **1.00** | 0.85 | 0.83 | 0.87 |
| N-ethylcarbazole | 0.68 | 0.99 | **1.00** | 0.01 | 0.96 | 0.91 |
| Pirimicarb | 1.04 | **1.00** | 0.46 | 0.89 | 0.84 | 0.78 |
| Progesterone | 2.47 | 0.98 | 0.97 | **1.00** | **1.00** | 0.87 |
| Quinoxaline | 0.78 | 0.94 | 0.96 | **1.00** | 0.93 | 0.88 |
| Tebuconazole | 5.28 | 0.95 | **1.00** | 0.77 | 0.91 | 0.92 |
| Testosterone | 6.30 | **1.00** | 0.98 | 0.65 | 0.79 | 0.87 |
| trans-Chalcone | 0.86 | **1.00** | **1.00** | 0.85 | 0.92 | 0.80 |
| Triclocarban | 10.97 | 0.86 | 0.81 | **1.00** | 0.87 | 0.86 |
| *Median* |  | **1.00** | **0.96** | **0.77** | **0.82** | **0.77** |
| *RSD [%]* |  | **10.1** | **19.5** | **52.8** | **20.4** | **16.8** |
| *Average rank* *(*$\bar{r}$*)* |  | **1.8** | **2.5** | **3.6** | **3.3** | **3.7** |

^a)^ Average retention times (t_R_) [min] from triplicate analysis in DCM:acetone.


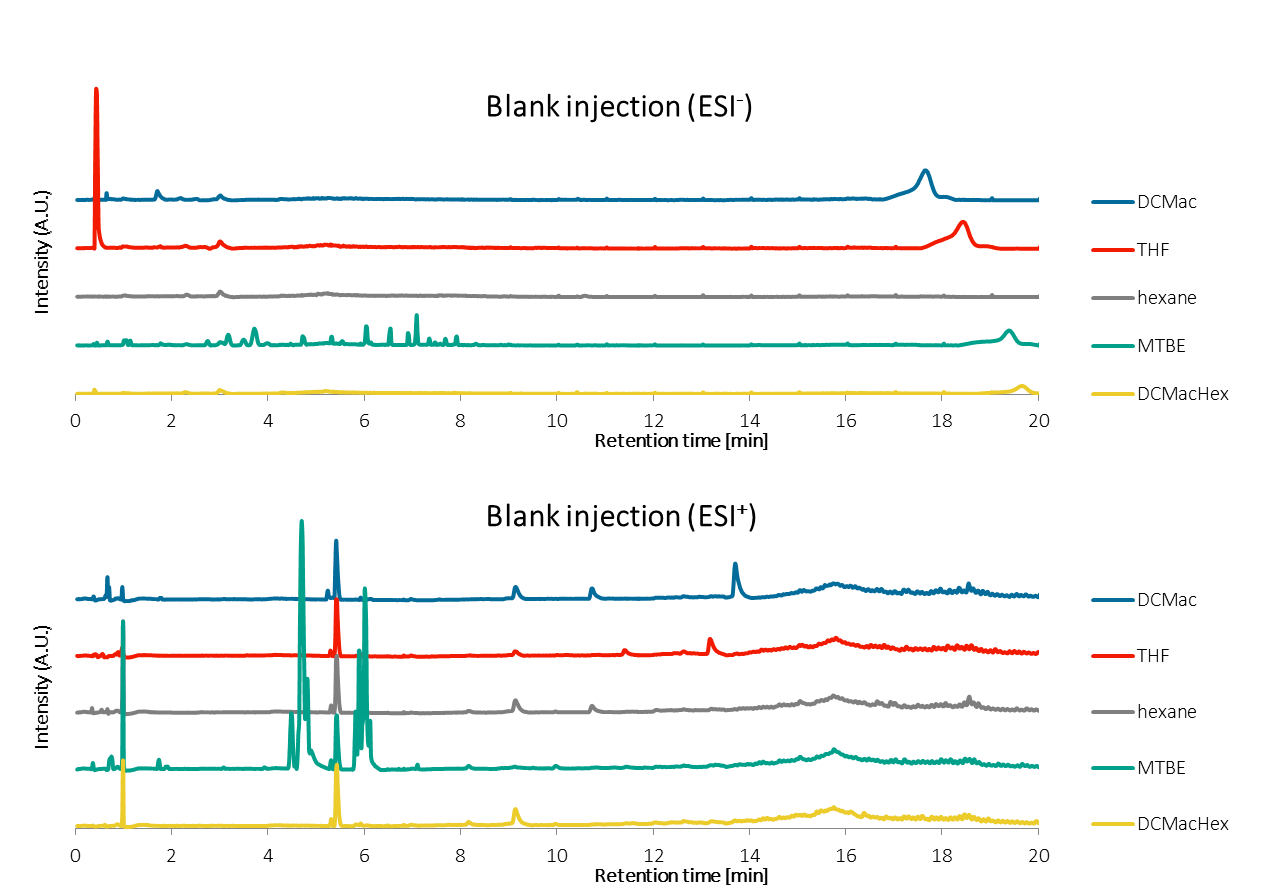


Figure S2. Blank injection of different injection solvents or mixtures. DCMac – DCM:acetone [3:1], THF – Tetrahydrofuran, MTBE – tert-methyl-butyl ether, DCMacHex – DCM:acetone:hexane [3:1:4]

**ESI^-^**

**ESI^+^**

Figure S3. Coefficients of *Design I* with peak capacity as the response for negative (ESI^-^) and positive ionisation (ESI^+^). FA – formic acid; AmAc – ammonium acetate; AmFo – ammonium formate; w – water addition; g – gradient steepness. * p ≤ 0.05; ** p ≤ 0.01; and *** p ≤ 0.001

Table S8. Validation experiments of *Design I* in ESI^+^.

| **ID** | **FA** | **AmAc** | **AmFo** | **w** | **g** | **Peak capacity** |
| --- | --- | --- | --- | --- | --- | --- |
| Val1 | 0 | +1 | 0 | -1 | -1 | 299 |
| Val2 | 0 | 0 | +1 | +1 | -1 | 313 |
| Val3 | 0 | 0 | +1 | +1 | -1 | 300 |
| Val4 | 0 | 0 | 0 | -1 | -1 | 211 |
| Val5 | 0 | +1 | 0 | +1 | -1 | 283 |


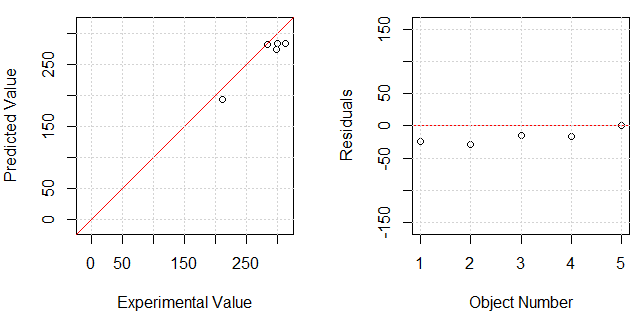


Figure S4. Prediction plots of peak capacities of the five validation experiments (projected onto the original model of *Design I*). Refer to Table S9 for further details.

Figure S5. Prediction plots (range-scaled areas) of *Design II*. (A) ESI- with ten validation experiments, (B) ESI+ with eleven validation experiments. Refer to Table S4 for details on validation experiments.

**A ESI^-^**

**B ESI^+^**

Table S9. Concentrations [ngg^-1^] in sediment samples after blank subtraction, incl. recoveries [%].

| **Concentration [ngg^-1^]** | **Recovery [%] ^a^** | **Fortress channel sediments (FSK) ^b^** | | | | | | | | | | | **Utterslev Mose lake sediments (UTM)** | | | | | | | | |
| --- | --- | --- | --- | --- | --- | --- | --- | --- | --- | --- | --- | --- | --- | --- | --- | --- | --- | --- | --- | --- | --- |
|  |  | **1C0** | **2C1** | **2C2** | **2C3** | **3C0** | **3C0a** | **6S0-1** | **6S0-5** | **7C0** | **8S0-2** | **8S0-3** | **11A** | **1F** | **1F-ax-1** | **1G** | **1Ga** | **1S** | **4L** | **5P** |  |
| 1,7-Dihydroxy­naphthalene | 27.8±19.0 | **0.77** | **<**LOQ | **1.22** | **0.91** | **8.58** | **2.69** | <LOQ | <LOQ | **6.01** | **2.41** | **<**LOQ | **4.19** | **2.53** | **<**LOQ | **<**LOQ | <LOQ | **1.09** | <LOQ | <LOQ |  |
| 1-Adamantanecarboxylic acid | 82.1±15.3 | ND | ND | ND | ND | ND | ND | ND | ND | ND | ND | ND | ND | ND | ND | ND | ND | ND | ND | ND |  |
| 1H-Benzo(g)indole | 66.6±10.1 | **1004.81** | **59.05** | **71.72** | **438.01** | **172.64** | **129.67** | **76.46** | **404.12** | **48.88** | **298.31** | **162.48** | **<**LOQ | **<**LOQ | <LOQ | <LOQ | <LOQ | <LOQ | <LOQ | <LOQ |  |
| 1-Hydroxy-2-naphthoic acid | 32.2±4.5 | **1.01** | **35.30** | <LOQ | <LOQ | **88.44** | **19.39** | <LOQ | **16.27** | **43.68** | **42.09** | **<**LOQ | **27.25** | **4.98** | <LOQ | <LOQ | <LOQ | <LOQ | <LOQ | <LOQ |  |
| 1-Naphthoic acid | 75.3±11.4 | **16.99** | **32.51** | **61.70** | **28.26** | **11.08** | **8.00** | <LOQ | **18.60** | **7.87** | **9.99** | <LOQ | <LOQ | **<LOQ** | <LOQ | <LOQ | <LOQ | <LOQ | <LOQ | <LOQ |  |
| 1-Pyrenecarboxylic acid | 54.3±8.1 | **4.55** | **24.41** | **19.36** | **38.52** | **14.50** | **4.61** | **15.54** | **11.93** | **6.62** | **16.02** | **<**LOQ | **1.07** | **2.51** | **6.06** | **1.68** | **2.89** | **1.15** | <LOQ | <LOQ |  |
| 2-Mercaptobenzothiazole | 13.7±3.6 | ND | ND | ND | ND | ND | ND | ND | ND | ND | ND | ND | ND | ND | ND | ND | ND | ND | ND | ND |  |
| 2-Naphthoic acid | 78.4±12.1 | <LOQ | **11.77** | **15.73** | **15.29** | **12.04** | **5.21** | <LOQ | <LOQ | **4.91** | **5.53** | **8.40** | <LOQ | <LOQ | <LOQ | <LOQ | <LOQ | <LOQ | <LOQ | <LOQ |  |
| 2-Phenylphenol | 83.5±11.4 | ND | ND | ND | ND | ND | ND | ND | ND | ND | ND | ND | ND | ND | ND | ND | ND | ND | ND | ND |  |
| 4-Butylbenzoic acid | 72.9±12.5 | <LOQ | <LOQ | <LOQ | <LOQ | <LOQ | <LOQ | <LOQ | <LOQ | <LOQ | <LOQ | <LOQ | <LOQ | <LOQ | <LOQ | <LOQ | <LOQ | <LOQ | <LOQ | <LOQ |  |
| 9,10-Anthraquinone | NA | ND | ND | ND | ND | ND | ND | ND | ND | ND | ND | ND | ND | ND | ND | ND | ND | ND | ND | ND |  |
| 9-Hydroxyphenanthrene | 38.8±11.0 | **4.70** | **5.86** | **29.70** | **35.75** | **17.30** | **16.47** | **5.27** | **10.06** | **7.22** | **14.21** | **11.31** | **<**LOQ | **0.76** | **1.05** | <LOQ | <LOQ | <LOQ | <LOQ | <LOQ |  |
| Acetophenone | NA | ND | ND | ND | ND | ND | ND | ND | ND | ND | ND | ND | ND | ND | ND | ND | ND | ND | ND | ND |  |
| Acridine | 93.1±10.1 | **46.39** | **17.46** | **103.92** | **287.96** | **121.76** | **127.16** | **47.43** | **197.51** | **74.58** | **95.06** | **107.11** | **7.00** | **9.37** | **6.77** | **6.91** | **7.47** | **5.39** | **1.50** | **1.37** |  |
| Amitriptyline | 77.2±6.7 | **10.78** | **3.67** | **5.09** | **1.60** | **5.12** | **2.87** | **1.31** | **0.58** | **4.56** | **4.13** | **0.19** | **1.30** | **1.17** | **0.78** | **1.09** | **1.05** | **0.63** | **<**LOQ | **<**LOQ |  |
| Anisole | 90.1±13.7 | <LOQ | <LOQ | <LOQ | <LOQ | <LOQ | <LOQ | **165.41** | **<**LOQ | <LOQ | <LOQ | <LOQ | <LOQ | <LOQ | <LOQ | **20.06** | **<**LOQ | **<**LOQ | **55.76** | <LOQ |  |
| Benzonitrile | 103.0±38.0 | <LOQ | <LOQ | **363.45** | <LOQ | <LOQ | <LOQ | **<**LOQ | **619.64** | <LOQ | <LOQ | <LOQ | <LOQ | <LOQ | <LOQ | <LOQ | <LOQ | <LOQ | <LOQ | <LOQ |  |
| Bisphenol A | 87.2±13.6 | **161.96** | **313.56** | **292.71** | **149.93** | **93.64** | **171.65** | **14.88** | **34.62** | **<**LOQ | **29.49** | **23.81** | **9.33** | **30.01** | **6.15** | <LOQ | <LOQ | <LOQ | <LOQ | <LOQ |  |
| Carbamazepine | 123.8±14.1 | **12.23** | **0.37** | **0.70** | **0.35** | **6.09** | **3.07** | **0.16** | **0.01** | **5.76** | **8.75** | **<**LOQ | **2.43** | **0.89** | **<**LOQ | **0.76** | **<**LOQ | **1.07** | **0.30** | <LOQ |  |
| Carbazole | 83.8±12.7 | **9.09** | <LOQ | <LOQ | <LOQ | <LOQ | <LOQ | <LOQ | <LOQ | <LOQ | **14.20** | <LOQ | <LOQ | <LOQ | <LOQ | <LOQ | <LOQ | <LOQ | <LOQ | <LOQ |  |
| Cyclohexanepentanoic acid | 74.0±15.5 | <LOQ | <LOQ | <LOQ | <LOQ | <LOQ | <LOQ | <LOQ | <LOQ | <LOQ | <LOQ | <LOQ | <LOQ | <LOQ | <LOQ | <LOQ | <LOQ | <LOQ | <LOQ | <LOQ |  |
| Cypermethrin | NA | ND | ND | ND | ND | ND | ND | ND | ND | ND | ND | ND | <LOQ | <LOQ | <LOQ | **1639.77** | **<**LOQ | <LOQ | <LOQ | <LOQ |  |
| Dibenzofuran | NA | ND | ND | ND | ND | ND | ND | ND | ND | ND | ND | ND | ND | ND | ND | ND | ND | ND | ND | ND |  |
| Dicyclohexylacetic acid | 78.4±19.5 | <LOQ | <LOQ | <LOQ | <LOQ | <LOQ | <LOQ | <LOQ | <LOQ | <LOQ | <LOQ | <LOQ | <LOQ | <LOQ | <LOQ | <LOQ | <LOQ | <LOQ | <LOQ | <LOQ |  |
| Diphenic acid | 48.8±8.3 | **<**LOQ | **19.38** | <LOQ | <LOQ | **34.15** | <LOQ | **<**LOQ | <LOQ | **138.46** | **<**LOQ | **<**LOQ | **3.71** | **4.89** | **3.25** | <LOQ | <LOQ | <LOQ | <LOQ | <LOQ |  |
| Estrone | 83.9±12.3 | <LOQ | <LOQ | **47.73** | **70.29** | **20.42** | <LOQ | **23.85** | <LOQ | **10.58** | **20.06** | **29.85** | <LOQ | <LOQ | <LOQ | <LOQ | <LOQ | <LOQ | <LOQ | <LOQ |  |
| Imazalil | 128.9±20.3 | **<**LOQ | **5.77** | **<**LOQ | **0.34** | **3.10** | **1.74** | **0.66** | **1.44** | **3.47** | **3.42** | <LOQ | **1.62** | **0.87** | **0.43** | **1.02** | **0.81** | **0.44** | **0.22** | **0.28** |  |
| Imidacloprid | 135.7±19.6 | <LOQ | <LOQ | <LOQ | <LOQ | **5.47** | **3.92** | <LOQ | <LOQ | **8.20** | **5.20** | <LOQ | **3.24** | <LOQ | <LOQ | <LOQ | <LOQ | <LOQ | <LOQ | <LOQ |  |
| Isoquinoline | 82.9±13.3 | **57.97** | **47.39** | **74.55** | **84.65** | **79.54** | **68.09** | **32.91** | **85.80** | **51.17** | **74.29** | **57.28** | **14.30** | **9.96** | **8.06** | **11.13** | **8.24** | **5.10** | **1.41** | **3.80** |  |
| Lauric acid | 61.6±14.9 | **2362.04** | **953.66** | **1088.82** | **3640.39** | **2213.85** | **5565.10** | **729.41** | **3120.94** | **822.27** | **3209.07** | **2830.69** | <LOQ | <LOQ | **70.95** | **<**LOQ | **35.06** | **50.83** | <LOQ | <LOQ |  |
| Linoleic acid | 55.4±6.2 | **2736.41** | **303.29** | **132.72** | **1275.61** | **382.64** | **555.63** | **153.36** | **169.33** | **176.40** | **135.21** | **665.26** | **607.62** | **412.58** | **939.05** | **583.76** | **1154.86** | **661.18** | **589.35** | **182.40** |  |
| Linolenic acid | 36.9±0.6 | **212.91** | **187.02** | **77.92** | **545.69** | **171.68** | **166.95** | **164.36** | **213.14** | **342.44** | **163.47** | **751.56** | **575.53** | **209.46** | **654.48** | **266.04** | **462.49** | **258.77** | **135.79** | **86.15** |  |
| N,N-Diethylacetamide | 85.3±22.7 | ND | ND | ND | ND | ND | ND | ND | ND | ND | ND | ND | ND | ND | ND | ND | ND | ND | ND | ND |  |
| N-Ethylcarbazole | 79.3±10.4 | ND | ND | ND | ND | ND | ND | ND | ND | ND | ND | ND | ND | ND | ND | ND | ND | ND | ND | ND |  |
| Oleic acid | 94.2±3.7 | **2622.90** | **777.97** | **464.90** | **4744.26** | **228.86** | **804.88** | **43.54** | **650.83** | **595.18** | **75.16** | **2128.45** | **89.63** | **2413.66** | **1038.25** | **507.44** | **1356.96** | **941.86** | **310.52** | **2.14** |  |
| Palmitoleic acid | 65.8±19.3 | **1570.71** | **1881.58** | **1228.29** | **1446.50** | **569.78** | **1249.24** | **1243.03** | **1524.47** | **1618.45** | **740.46** | **2273.97** | **427.16** | **553.85** | **2258.47** | **971.35** | **1730.08** | **1349.45** | **500.34** | **483.58** |  |
| Pentachlorophenol | 75.2±10.6 | **26.40** | **17.75** | **32.05** | **44.13** | **16.63** | **29.52** | **8.33** | **2.67** | **8.25** | **30.67** | **2.46** | **2.90** | **2.72** | **1.65** | **7.64** | **4.77** | **2.47** | **<**LOQ | **0.03** |  |
| PFOA | 82.5±15.1 | **0.77** | **1.40** | **0.71** | **0.32** | **8.87** | **0.14** | **0.21** | **<LOQ** | **5.07** | **0.22** | **<**LOQ | **2.04** | **1.46** | **<**LOQ | **0.59** | **0.07** | **0.73** | **<**LOQ | **<**LOQ |  |
| PFOS | NA | **5.17** | **4.26** | **2.60** | **4.64** | **8.06** | **0.60** | **0.33** | **0.36** | **5.39** | **0.45** | **0.20** | **2.56** | **1.66** | **0.58** | **1.86** | **1.27** | **1.29** | **0.73** | **0.37** |  |
| Phthalic acid | 27.8±19.0 | ND | ND | ND | ND | ND | ND | ND | ND | ND | ND | ND | ND | ND | ND | ND | ND | ND | ND | ND |  |
| Pirimicarb | 122.2±22.5 | **2.31** | **2.16** | **5.92** | **4.04** | **9.05** | **4.82** | **4.79** | **4.72** | **9.93** | **9.38** | **2.30** | **1.68** | **0.72** | **0.02** | **1.18** | **0.01** | **1.31** | **0.42** | **0.36** |  |
| Progesterone | 65.3±13.2 | **0.51** | **1.47** | <LOQ | <LOQ | **5.75** | **5.93** | **0.49** | **2.25** | **7.40** | **2.66** | **1.19** | **2.65** | **2.27** | **5.25** | **2.20** | **<**LOQ | **0.91** | <LOQ | <LOQ |  |
| Pyrocatechol | NA | ND | ND | ND | ND | ND | ND | ND | ND | ND | ND | ND | ND | ND | ND | ND | ND | ND | ND | ND |  |
| Quinoxaline | 88.7±16.5 | **16.71** | **16.26** | **30.20** | **47.24** | **44.11** | **19.03** | <LOQ | <LOQ | <LOQ | **25.28** | <LOQ | <LOQ | **7.26** | <LOQ | <LOQ | <LOQ | <LOQ | <LOQ | <LOQ |  |
| Salicylic acid | NA | ND | ND | ND | ND | ND | ND | ND | ND | ND | ND | ND | ND | ND | ND | ND | ND | ND | ND | ND |  |
| Tebuconazole | 134.5±19.1 | **2.28** | **2.94** | **0.57** | **<**LOQ | **4.77** | **3.64** | **0.95** | **<**LOQ | **4.68** | **5.18** | <LOQ | **2.36** | **2.04** | **0.92** | **1.38** | <LOQ | **1.57** | **0.36** | **<**LOQ |  |
| Testosterone | 88.6±13.1 | **0.41** | **1.05** | **0.27** | **2.13** | **5.86** | **3.06** | **1.05** | **2.74** | **5.34** | **7.83** | <LOQ | **2.42** | **1.77** | **<**LOQ | **1.64** | <LOQ | **1.45** | **<**LOQ | **0.71** |  |
| trans-Chalcone | 79.8±12.1 | **107.09** | **52.43** | **129.90** | **198.15** | **116.05** | **114.33** | **68.50** | **154.13** | **83.39** | **132.89** | **94.77** | **11.68** | **2.69** | **1.18** | **53.97** | **33.32** | **23.27** | **<**LOQ | **<**LOQ |  |
| Triclocarban | 66.5±9.3 | **1193.04** | **314.99** | **1405.83** | **409.39** | **304.17** | **353.06** | **122.98** | **59.64** | **326.76** | **254.97** | **76.39** | **24.25** | **98.16** | **86.33** | **113.34** | **120.25** | **11.78** | **5.75** | **1.24** |  |
| Triclosan | 89.8±15.6 | **97.46** | **41.92** | **44.24** | **40.35** | **30.48** | <LOQ | <LOQ | <LOQ | <LOQ | **44.51** | <LOQ | <LOQ | <LOQ | <LOQ | <LOQ | **14.62** | <LOQ | <LOQ | <LOQ |  |

^a)^ *NA* – not available. Recovery experiments were run with the QC method (*cf*. Table S6). *ND* – not detected; <*LOQ* – below limit of quantification (viz. SN < 10).

^b)^ All samples cover 0-30 cm in depth. 2C1, 2C2 and 2C3 are in-depth profiles covering 0-10, 0-20 and 0-30 cm, respectively.
